# Gut Microbiota and Atherosclerotic Plaque Instability: Cellular and Molecular Mechanisms

**DOI:** 10.3390/ijms27136001

**Published:** 2026-07-03

**Authors:** Riccardo Nieri, Martina Pitolli, Matteo Antonio Russo, Federica Limana

**Affiliations:** 1 Department of Experimental Medicine, Sapienza University of Rome, 00161 Rome, Italy; riccardo.nieri@uniroma1.it; 2 Technoscience, Parco Scientifico e Tecnologico Pontino, 04100 Latina, Italy; martina.pitolli@gmail.com; 3 IRCCS San Raffaele Roma, 00166 Rome, Italy; matteoantoniorusso44@gmail.com; 4 Dipartimento di Promozione delle Scienze Umane e della Qualità della Vita, San Raffaele University of Rome, 00166 Rome, Italy; 5 Laboratorio di Patologia Cellulare e Molecolare, IRCCS San Raffaele Roma, 00166 Rome, Italy

**Keywords:** atherosclerosis, gut microbiota, plaque vulnerability, vascular inflammation, microbial metabolites, cardiovascular disease

## Abstract

Atherosclerosis is a chronic multifactorial inflammatory vascular disease and the major risk factor for cardiovascular diseases (CVDs), characterized by arterial wall thickening, loss of arterial elasticity and the progressive accumulation of lipids and immune cells, ultimately leading to plaque formation and the development of unstable, rupture-prone plaques. Several studies suggest that gut microbiota might contribute to atherosclerosis, mainly by converting dietary and endogenous molecules into bioactive metabolites, such as trimethylamine N-oxide (TMAO), short-chain fatty acids (SCFAs), and the Gram-negative cell-wall component lipopolysaccharide (LPS). Such metabolites can promote key mechanisms involved in the development and progression of atherosclerotic plaque, and, importantly, plaque vulnerability. Specifically, they can worsen endothelial dysfunction, induce macrophage-driven inflammatory responses, and cause oxidative stress and extracellular matrix degradation. These processes are crucial for thinning of the fibrous cap and destabilization of atherosclerotic plaques. As a result, the risk of plaque rupture and related cardiovascular events increases. In this review, we summarize potential mechanisms by which the gut microbiota and their compounds induce the formation of vulnerable atherosclerotic plaques and discuss findings from experimental models and clinical studies that reveal the crucial role of microbiota–host dynamics in atherosclerosis. In contrast to previous reviews that primarily focused on atherosclerosis development, we specifically highlight the cellular and molecular mechanisms linking gut microbiota to plaque vulnerability and destabilization. We also address future research priorities to define microbiota-driven pathways better and develop targeted therapeutic interventions to reduce plaque vulnerability and cardiovascular risk.

## 1. Introduction

Atherosclerosis is a chronic inflammatory disease of the arterial wall, characterized by the formation of fibrous plaques within medium- and large-diameter arteries, primarily resulting from lipid deposition in the arterial wall, which subsequently triggers an inflammatory response [1]. The condition generally remains asymptomatic until it reaches advanced stages, when plaque rupture may precipitate severe complications such as myocardial infarction (MI) and stroke [2].

Recent research has highlighted the significant role of the gut microbiota in the pathogenesis and progression of different cardiovascular diseases (CVDs) [3].

The gut microbiota mainly comprises diverse bacterial taxa, while archaea, fungi, viruses, and other microbial entities contribute to a minor degree [4]. The primary bacterial phyla, Bacteroidetes and Firmicutes, exhibit variable abundance influenced by diet, geographic location and lifestyle factors [5,6].

This complex system is highly individualized since both microbial composition and metabolic activity can vary among individuals and over time. Given this complexity and interindividual variability, dysbiosis of the gut microbiota has been implicated in the development and progression of several CVDs, including hypertension, atherosclerosis, and heart failure [7].

Specifically, bacteria influence atherosclerosis through several mechanisms, including the translocation of inflammatory microbial components such as lipopolysaccharide (LPS) from the intestinal lumen into the circulation due to increased gut permeability, modulation of lipid metabolism, increased production of harmful metabolites such as trimethylamine N-oxide (TMAO), and decreased synthesis of beneficial metabolites such as short-chain fatty acids (SCFAs). All these mechanisms significantly impact vascular health [8,9]. Importantly, stable plaques remain pathological lesions; however, increased plaque stability is generally associated with a lower risk of rupture and acute cardiovascular events compared to vulnerable plaques [10,11,12].

Although the association between gut microbiota and atherosclerosis has been extensively investigated, the specific mechanisms linking microbiota-derived signals to plaque vulnerability remain incompletely understood [10,11,12]. This review aims to provide an integrated overview of the cellular and molecular mechanisms through which gut microbiota and their metabolites influence atherosclerotic plaque instability. Particular attention is given to the effects of microbiota–host interactions on inflammation, macrophage polarization, efferocytosis, extracellular matrix remodeling, and other pathways involved in the transition from stable to vulnerable plaques. We also summarize evidence from experimental models and clinical studies supporting the role of the gut microbiota–vascular axis in plaque destabilization and discuss its potential therapeutic implications.

## 2. Atherosclerosis: Pathogenesis and Determinants of Plaque Vulnerability

Ischemic disease is currently the most important cause of heart failure, which is considered a major public health problem. However, ischemic heart disease is the most common cause of cardiovascular death, accounting for 38% of all CVD deaths in females and 44% in males. Consequently, the best prevention for this type of heart disease is to reduce recurrent episodes of coronary instability [13]. Recent reports indicate that CVDs accounted for 32% of global deaths in 2021 [14,15].

Atherosclerosis is a chronic inflammatory disease whose progression can be related to, or unrelated to, atheroma plaque formation [2]. In the former case, the arteries involved show ectasia (vessel dilatation), which can progress to an aneurysm or aortic dissection. In the other case, atherosclerotic plaques induce vessel obstruction, with consequent CVD, including coronary artery disease (CAD), stroke, angina pectoris, or Peripheral Vascular Disease (PVD) [16]. Atherosclerosis is largely asymptomatic until the late stages of the disease, which occur decades later, when plaque rupture and thrombosis may trigger a range of clinical manifestations, with myocardial infarction (MI) and cerebrovascular accident (CVA) being the most common [2].

Atherogenesis is influenced by a range of risk factors, including hypercholesterolemia, hypertension, diabetes, smoking, obesity, and chronic inflammation [16]. However, hypercholesterolemia is the major risk factor for the development of atherosclerosis [17]. Elevated circulating low-density lipoprotein (LDL) levels increase the risk of oxidation and accumulation within the arterial wall, leading to atherogenesis. When LDL is oxidized by locally produced reactive oxygen species (ROS), it forms oxidized LDL (oxLDL), which contributes to the immunological response in atherosclerosis through receptors such as Cluster of Differentiation 36 (CD36), Scavenger Receptor class A (SR-A), and Lectin-like oxidized low-density lipoprotein receptor-1 (LOX-1) [18,19]. This oxLDL then stimulates surrounding endothelial cells (ECs) through LOX-1 [20], as well as through uptake by trans-endothelial transport or diffusion at cell–cell junctions determined by disturbed blood flow, leading to increased expression of adhesion molecules such as P-selectin, E-selectin, VCAM1, and ICAM1, and to the release of pro-inflammatory mediators such as chemokines and cytokines, including IL-1, IL-8, monocyte chemoattractant protein-1 (MCP-1), granulocyte–monocyte stimulating factor, and NF-κB [16,18,21].

This sequence induces infiltration of circulating immune cells, including monocytes and T lymphocytes, via increased expression of adhesion molecules and chemotactic mediators, and promotes vascular smooth muscle cell (VSMC) proliferation, migration, phenotypic switching, foam cell formation, and platelet activation. During early atherosclerosis, macrophages engulf oxLDL, which induces the release of several pro-inflammatory cytokines such as tumor necrosis factor alpha (TNF-α), IL-1β, IL-6, IL-12, and IL-23, and facilitates the deregulation and excessive uptake of modified LDL, leading to the formation of lipid-laden foam cells. The initial accumulation of foam cells in the tunica intima of the vessel wall is responsible for early lesion formation, referred to as a “fatty streak” [22]. Abnormal intracellular cholesterol loading in macrophage-derived foam cells induces cellular stress responses, including endoplasmic reticulum stress and oxidative stress, ultimately leading predominantly to apoptosis. However, other forms of regulated cell death, such as pyroptosis, necroptosis, ferroptosis, and erythrophagocytosis, may also contribute to plaque progression. Dying foam cells release pro-inflammatory mediators, including TNF-α, interleukin (IL)-1β, and IL-6, which amplify local inflammation, promote immune cell recruitment, and contribute to endothelial dysfunction. Continued cell death, together with defective efferocytosis, leads to the formation of a hypocellular necrotic core composed of dead cells, apoptotic bodies, cellular debris, and extracellular cholesterol deposits [23]. As plaques progress, the hypoxic and hemorrhagic microenvironment further promotes cell death and expansion of the necrotic core, while cholesterol crystals can activate inflammatory pathways, including the NLRP3 inflammasome, thereby perpetuating chronic inflammation and plaque destabilization. Furthermore, a fibrous cap forms, encapsulating and stabilizing the plaque. Fibrous cap formation coincides with the infiltration of VSMCs (from the tunica media) into the intima; VSMCs undergo a phenotypic shift from a quiescent state to a proliferative one and produce extracellular matrix (ECM) proteins such as collagens, elastin, and proteoglycans, which become major constituents of the fibrous cap [24].

In physiological conditions, apoptotic cells are rapidly cleared by phagocytes through efferocytosis. In advanced atherosclerotic lesions, however, efferocytosis becomes defective due to impaired recognition and engulfment of apoptotic cells by macrophages, resulting in the accumulation of uncleared apoptotic cells that undergo secondary necrosis. This process promotes the release of pro-inflammatory mediators, enhances immune cell recruitment, and sustains chronic plaque inflammation [25]. Defective efferocytosis contributes to the persistence of a pro-inflammatory macrophage phenotype and hampers the resolution of inflammation within the plaque [25]. In advanced stages of atherosclerosis, lesional macrophages and other cells secrete pro-inflammatory molecules, including matrix metalloproteinases (particularly MMP-2 and MMP-9) and other proteases, such as cathepsins, which promote extracellular matrix degradation and weakening of the fibrous cap [26]. Fibrous cap thinning is further accelerated by reduced expression of tissue inhibitors of metalloproteinases (TIMPs) and sustained inflammation, which impairs VSMC survival and promotes VSMC apoptosis, thereby limiting the reparative capacity of the plaque [27].

According to their morphological and biological features, atherosclerotic plaques may be classified as stable or unstable [28]. Stable plaques are generally characterized by a thick fibrous cap, abundant collagen content, preserved VSMC abundance, and relatively low inflammatory cell infiltration. In contrast, unstable plaques exhibit a large lipid-rich necrotic core, a thin fibrous cap, increased inflammatory cell accumulation within the cap, intraplaque hemorrhage, neovascularization, calcification, elevated protease activity, reduced collagen content, and a loss of VSMCs due to apoptosis [29,30]. These features increase the susceptibility of plaques to rupture and subsequent thrombotic events, ultimately leading to ischemia or plaque erosion [31].

### 2.1. Cellular Determinants of Plaque Vulnerability

**Figure 1:**
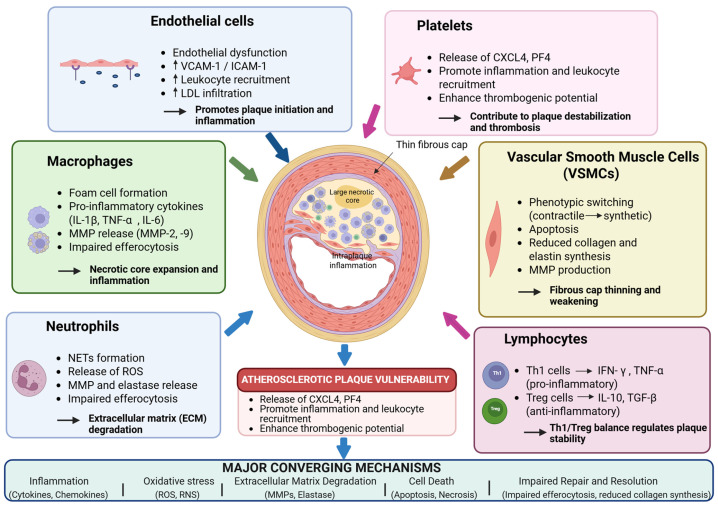
Cellular determinants of atherosclerotic plaque vulnerability.

The development of plaque vulnerability results from coordinated interactions among endothelial cells, vascular smooth muscle cells, inflammatory cells, and platelets. The principal cellular mechanisms involved in plaque destabilization are summarized in Figure 1.

Endothelial cells (ECs), macrophages, neutrophils, vascular smooth muscle cells (VSMCs), platelets, and lymphocytes contribute to plaque vulnerability through interconnected mechanisms that promote inflammation, oxidative stress, extracellular matrix (ECM) degradation, cell death, and impaired tissue repair. Endothelial dysfunction enhances leukocyte recruitment and low-density lipoprotein (LDL) infiltration. Macrophages promote foam cell formation, inflammation, and necrotic core expansion through the release of pro-inflammatory cytokines and matrix metalloproteinases (MMPs). Neutrophils contribute to plaque destabilization by releasing reactive oxygen species (ROS), MMPs, and neutrophil extracellular traps (NETs), which amplify inflammation and extracellular matrix degradation. VSMCs undergo phenotypic switching from a contractile to a synthetic phenotype, which is characterized by reduced contractile function and enhanced extracellular matrix (ECM) remodeling. Together with VSMC apoptosis, these changes contribute to fibrous cap thinning and weakening. Activated platelets enhance inflammation and thrombogenicity through the release of platelet factor 4 (PF4/CXCL4), CD40 ligand (CD40L), and other pro-inflammatory mediators. The balance between pro-inflammatory T helper 1 (Th1) cells and anti-inflammatory regulatory T (Treg) cells further influences plaque stability. These converging cellular mechanisms ultimately determine the development of a vulnerable plaque phenotype characterized by a large necrotic core, a thin fibrous cap, increased inflammatory cell content, and an elevated risk of plaque rupture and thrombosis.

#### 2.1.1. Endothelial Cells (ECs)

ECs play a key role in regulating local vascular tone and maintaining vascular homeostasis [32]. In the presence of disturbed flow, ECs and their tight junctions become “leaky”, lose their characteristic flattened shape and elongated alignment in the direction of flow, and increase their volume by adopting a cobblestone appearance [32]. This event promotes the uptake of plasma LDL and triglyceride (TG)-rich lipoproteins via transendothelial transport or paracellular diffusion through disrupted intercellular junctions [16]. In lesion-prone arterial regions, the actions of pro-inflammatory mediators such as IL-1, tumor necrosis factor alpha (TNF-α), endotoxin, oxLDL, and advanced glycation end products (AGEs), as well as biomechanical stimulation from disturbed blood flow, lead to EC activation. These biochemical and biomechanical stimuli predominantly signal via the pleiotropic transcription factor nuclear factor-κB (NF-κB). They result in a coordinated genetic regulatory program within ECs, including the expression of adhesion molecules (CAMs) on the cell surface as vascular cell adhesion molecule-1 (VCAM-1), endothelial cell adhesion molecule (ICAM-1), endothelial (E) selectin, platelet (P) selectin, and the release of chemokines, like monocyte chemoattractant protein (MCP)-1, and fractalkine. ECs also express prothrombotic mediators such as plasminogen activator inhibitor (PAI)-1 and endothelial transforming growth factor-beta (TGF-β) [33], resulting in the selective recruitment of monocytes and various T lymphocyte subsets, which become resident in the subendothelial space. In addition, disturbed blood flow induces EC dysfunction via toll-like receptor (TLR) 2-dependent apoptosis and consequent secretion of IL-8, leading to neutrophil recruitment [16], and increases EC permeability to large molecules through multiple pathways, including BMP-TGF-β, WNT, and Notch [34]. The concerted actions of activated endothelial cells, smooth muscle cells, monocyte/macrophages, and lymphocytes produce a complex paracrine milieu. This milieu contains cytokines, growth factors, and ROS within the vessel wall. These factors perpetuate chronic inflammation by promoting continuous immune cell recruitment, endothelial activation, oxidative stress, and extracellular matrix remodeling, thereby driving atherosclerotic lesion progression [21]. In addition, mitochondrial dysfunction and oxidative stress have emerged as important contributors to endothelial senescence and vascular injury during the progression of atherosclerosis, further exacerbating endothelial dysfunction and inflammatory signaling [35]. On the other hand, in response to signals such as TGF-β, ECs can transdifferentiate into a mesenchymal phenotype via endothelial-to-mesenchymal transition, a process that may be further enhanced by inflammatory stimuli and that contributes to vascular remodeling, plaque progression, and plaque vulnerability [36].

#### 2.1.2. Macrophages

Increased expression of adhesion molecules (e.g., VCAM-1 and ICAM-1) and chemotactic proteins (e.g., MCP-1) in activated ECs recruits monocytes from the blood to the vessel wall. Upon entering the intima, monocytes differentiate into macrophages in response to locally produced Mac colony-stimulating factor (M-CSF) and related cytokines such as IFN-γ, IL-4, or IL-13 [16]. The lesion microenvironment affects the differentiation of monocytes into macrophages. Macrophages phagocytize the modified lipoproteins, become foam cells, and are retained within plaques. Consequently, more monocytes are recruited to the lesion area, contributing to necrotic core formation. Interestingly, macrophages can polarize into pro- and anti-inflammatory subtypes, exacerbating or alleviating disease [22]. Different macrophage subclasses have been identified in plaques, including M1, M(Hb), Mhem, M2, Mox, and M4. These macrophage phenotypes represent a dynamic continuum rather than fixed populations, and transitions between activation states may occur in response to local microenvironmental cues, including lipid accumulation, cytokines, oxidative stress, and microbial-derived signals [37].

M1 macrophages in atherosclerotic lesions can be stimulated by cholesterol crystals, lipopolysaccharide (LPS), and pro-inflammatory cytokines such as IL-18, and oxLDL. They are normally activated by TLR-2, -4, and -6 or by NF-κB, and secrete pro-inflammatory cytokines such as TNF-α, IL-1β, IL-6, IL-12, and IL-23 [37].

M2 macrophages exist in three subtypes. M2a, activated by IL-4 and IL-13, is shown to be beneficial in atherosclerosis. M2c, activated by glucocorticoids and TGF-β, phagocytizes debris and apoptotic cells. In contrast, M2b is induced by immune complexes, IL-1b, and LPS. M2b expresses TGF-β but also produces inflammatory cytokines such as IL-1β, IL-6, and TNF-α [37]. M(Hb) and Mhem macrophages are anti-inflammatory and produce cytokines like IL-10. Conversely, Mox macrophages and M4 macrophages are pro-inflammatory, producing IL-1β, IL-6, TNF-α, and MMPs. Notably, Mox macrophages comprise 30% of total macrophages in progressive plaques [37].

The current literature suggests M1 macrophages accumulate more lipids than M2 macrophages. M1 macrophages induce inflammation, have lower fatty acid oxidation, increased glycolytic metabolism (especially anaerobic glycolysis and the pentose phosphate pathway), enhanced fatty acid synthesis, and elevated ROS production. In contrast, M2 macrophages show more active fatty acid oxidation and fatty acid consumption with increased ATP production through the electron transport chain and oxidative phosphorylation, along with lower levels of glycolysis [37].

#### 2.1.3. Vascular Smooth Muscle Cells (VSMCs)

VSMCs originate from multiple progenitor cells. For example, VSMCs from the ascending aorta, carotid arteries, and arch originate from the neuronal crest, while VSMCs from the descending aorta predominantly derive from somatic precursors [38]. In atherosclerotic plaques, VSMCs can represent 30–70% of total cells and contribute to the necrotic core and the fibrous cap [39]. The origin of VSMCs in plaques remains unclear. Most studies indicate that VSMCs migrate from the tunica media to the tunica intima, with other sources including adventitial mesenchymal stem cells, pericytes, fibroblasts, and ECs [31,38,40]. VSMCs regulate arterial contractions and resistance, produce ECM proteins (elastin, collagen, proteoglycans), and are crucial for vascular compliance and arterial elastic recoil in response to hemodynamic changes [31]. VSMCs can undergo several phenotypic switches controlled by major regulatory pathways, including TGF-β, NF-κB, KLF4, OCT4, and PDGF-BB. In particular, VSMCs can switch from a contractile to a synthetic phenotype, characterized by reduced expression of contractile markers, together with enhanced proliferative, migratory, and extracellular matrix-producing capacity. In addition, VSMCs may acquire foam-cell-like, macrophage-like, mesenchymal-like, endothelial-like, myofibroblast-like, and osteochondrogenic phenotypes [38]. The VSMC phenotypic switch impacts plaque stability. Foam-cell-like and macrophage-like VSMCs are generally associated with lipid accumulation, inflammation, and plaque progression. In contrast, mesenchymal-like and myofibroblast-like VSMCs may contribute to extracellular matrix deposition, fibrous cap formation, and plaque stabilization. The contribution of other VSMC phenotypes to plaque stability remains less clearly defined [38]. VSMCs are pivotal in the pathogenesis of plaque rupture and erosion. VSMC death or senescence reduces ECM deposition, increases pro-inflammatory cytokines and MMPs, and drives plaque rupture. Altered ECMs from VSMCs, rich in hyaluronic acid, type III collagen, and versican, can degrade hyaluronic acid, activating ECs via TLR2. This process induces EC apoptosis, neutrophil recruitment, and platelet activation [31].

#### 2.1.4. Neutrophils

Neutrophils are the most abundant white blood cells in human circulation and the primary cell type involved in acute inflammation. Neutrophils originate from the bone marrow, and their release into the bloodstream is modulated by chemokines, adhesion molecules, and growth factors, while chemotactic molecules, such as CC-chemokine receptor 1, 2, and 5 (CCR1, CCR2, CCR5) and CXC-chemokine receptor 2 (CXCR2), drive their recruitment to areas of interest [41].

The number of neutrophils in the intima of the arteries is related to plaque instability [42]; yet, higher levels of neutrophils are present in the fibrous cap area prone to rupture than in stable lesions [43]. Furthermore, circulating neutrophils positively correlate with the size of developing lesions [44].

Briefly, during atherogenesis, adhesion molecules such as P-selectin and E-selectin expressed by ECs promote the recruitment of neutrophils into the plaque area [45]. Activated ECs recruit neutrophils through selectins and chemokines, whereas neutrophil-derived ROS, proteases, and NETs further enhance endothelial activation and dysfunction, creating a self-amplifying inflammatory loop within the plaque [45,46]. Neutrophils, in turn, secrete granule proteins such as cathepsin G and cathelicidin, which promote the recruitment of additional neutrophils and circulating monocytes to the developing lesion. Once recruited, these monocytes differentiate into macrophages, whose activation is further enhanced by neutrophil-derived mediators, including cathelicidin and α-defensin. In the lumen and intima, neutrophils release proteases and ROS, resulting in endothelial dysfunction, with consequent ECM deposition and increased LDL extravasation. In addition, neutrophils are involved in the progression of atherogenesis by: (I) activating macrophages through the release of cathelicidin and a-defensin (II), promoting foam cell formation by the oxidation of LDL in oxLDL through myeloperoxidase (MPO), an (III) releasing neutrophil extracellular traps (NETs) which stimulate plasmacytoid dendritic cells (pDCs) to produce interferon-a (IFN-a) and macrophages to produce IL-1b and IL-18. During the late stages of atherosclerosis, neutrophils can destabilize the plaque by secreting NETs containing cytotoxic histone H4, which perforates and eventually lyses vascular smooth muscle cells (VSMCs). VSMC death is also induced by neutrophil-derived MMPs, which degrade the ECM. VSMC death and ECM degradation result in thinning of the fibrous cap and the formation of rupture-prone vulnerable plaques [46].

#### 2.1.5. Platelets

Platelets are anuclear bood cells involved in the development and progression of atherosclerosis and play a crucial role in inflammation and thrombosis. Platelets may be activated upon adhesion to ECs in response to the expression of adhesion molecules, such as von Willebrand Factor (vWF), ICAM-1, and fibronectin, or by thrombogenic molecules, such as ADP (adenosine diphosphate), collagen, and thrombin [47]. Platelet activation induces the release, in the circulation and in the local environment, of pro-inflammatory cytokines and chemokines such as CXCL4 or platelet factor 4 (PF4), which play a crucial role in atherosclerosis, and MMPs such as MMP-2 and MMP-9, which contribute to extracellular matrix degradation and local activation of factors favoring plaque rupture and atherothrombosis [47].

#### 2.1.6. Lymphocytes

Lymphocytes are involved in atherosclerosis; briefly, the most abundant T cell types in the lesion are TH1 cells, which promote plaque growth and instability by producing interferon-γ, and Treg and TH2 cells, which exert protective effects by releasing IL-10, TGF-β, IL-5, and IL-15. T cells may be recruited by chemokine receptors CCR5 and CXCR6. B cells may contribute to the development and progression of atherosclerosis by producing antibodies that bind specific antigens or by driving an autoimmune response. Accordingly, antibodies against oxLDL and LDL, as well as elevated immunoglobulin E (IgE) levels, are significantly associated with atherosclerosis [16].

## 3. Influence of Gut Microbiota on Plaque Instability

### 3.1. Gut Microbiota and Atherosclerotic Plaque Instability

**Figure 2:**
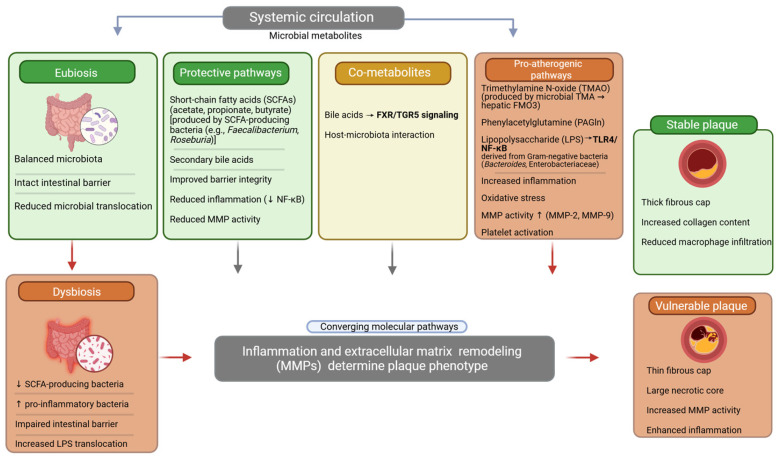
Gut microbiota–vascular axis in atherosclerotic plaque stability and instability.

The relationship between gut microbiota and atherosclerotic plaque instability has been shown to be an important subject of research [48,49]. Several studies have demonstrated that gut microbiota may influence the stability of atherosclerotic plaques by synthesizing specific metabolites, regulating inflammatory pathways, modulating the immune system, altering plaque composition, and impairing intestinal barrier integrity [10,11,12]. In the following paragraphs, these mechanisms will be examined using recent research findings (Figure 2).

In the presence of eubiosis, beneficial microbial taxa, including Faecalibacterium and Roseburia, promote the formation of metabolites such as short-chain fatty acids (SCFAs) and secondary bile acids, which improve intestinal barrier integrity, reduce metalloproteinase (MMP) activity, and suppress pro-inflammatory signaling pathways such as nuclear factor-κB (NF-κB). In contrast, dysbiosis, characterized by a reduced abundance of beneficial bacteria and an increased prevalence of pro-inflammatory microorganisms, favors microbial translocation and the production of molecules such as trimethylamine N-oxide (TMAO) and phenylacetylglutamine (PAGln). Gram-negative bacteria, including members of the Bacteroides genus and the Enterobacteriaceae family, contribute to circulating lipopolysaccharide (LPS), a bacterial cell-wall component, which activates inflammatory and oxidative stress pathways, including the Toll-like receptor 4 (TLR4)/NF-κB signaling pathway. Bile acids act as key co-metabolites mediating host–microbiota interactions through activation of nuclear and membrane receptors, including farnesoid X receptor (FXR) and Takeda G protein-coupled receptor 5 (TGR5). These converging molecular pathways regulate inflammation and extracellular matrix remodeling, including MMP activity, which ultimately determines the plaque phenotype. Protective signaling is associated with increased collagen content, reduced macrophage infiltration, and the formation of stable plaques with thick fibrous caps. In contrast, pro-atherogenic signaling promotes inflammation, increased MMP activity, and the formation of vulnerable plaques with a thin fibrous cap and large necrotic cores.

### 3.2. Metabolites and Their Role in Plaque Instability

#### 3.2.1. Trimethylamine N-Oxide (TMAO)

TMAO is generated through the oxidation of gut-microbiota-derived trimethylamine (TMA) by hepatic flavin-containing monooxygenases. The gut microbiota, via dietary phosphatidylcholine and L-carnitine, generates TMA. Elevated levels of TMAO have been associated with increased cardiovascular risk in several clinical studies [50,51]. Notably, the relationship between TMAO levels and the extent of atherosclerotic plaque remains controversial [52,53]. At the same time, several studies have demonstrated that TMAO impairs plaque stability by promoting macrophage foam cell formation and by increasing the expression of pro-inflammatory cytokines, thereby destabilizing the plaque’s fibrous cap [48,54,55].

Using two well-accepted mouse models of plaque instability (ApoE^−/−^ and Ldlr^−/−^ mice), which develop hypercholesterolemia and accelerated atherosclerotic lesions due to impaired lipoprotein clearance, Koay and colleagues detected fold elevations of plasma TMA and TMAO in animals fed a 3% choline-supplemented diet compared with those receiving a 0.3% choline-supplemented high-fat, high-cholesterol diet, without significant changes in plaque burden or composition. This finding was corroborated by results from the Framingham Heart Study, which showed no correlation between TMAO levels and atherosclerotic progression. Nevertheless, using the tandem-stenosis mouse model, a validated model of plaque vulnerability based on flow-disturbance-induced plaque destabilization, they demonstrated a significant association between TMAO plasma levels and various characteristics of plaque instability, including inflammatory markers, platelet activation, and intraplaque hemorrhage [55].

Another recent study investigated the mechanisms underlying TMAO’s proatherogenic influence and its contribution to plaque instability. By single-cell RNA-sequencing analysis on aortic atherosclerotic regions of female Ldlr^−/−^ mice under TMAO feeding for 3 months, Cheng and collaborators revealed that TMAO supplementation promoted VSMC apoptosis and decreased collagen and ECM content. TMAO also exerted direct effects on VSMC-derived macrophages, decreasing collagen production and increasing collagen degradation via macrophage-released MMPs, therefore affecting plaque stability, i.e., decreasing fibrous cap thickness and collagen deposition [56].

#### 3.2.2. Short-Chain Fatty Acids (SCFAs)

SCFAs are organic acids produced by the fermentation of dietary fibers, including resistant starch, inulin, and pectin, by SCFA-producing bacteria such as *Faecalibacterium*, *Roseburia*, and *Eubacterium* species. They are characterized by a short carbon chain (C1–C6), with acetate (C2), propionate (C3), and butyrate (C4) being the most abundant and extensively studied SCFAs in human metabolism [57]. About 90% of the SCFAs present in the intestinal lumen are absorbed by the apical surface of colonocytes via solute carrier family 26 member 3 (SLC26M3) and monocarboxylate transporter 1 (MCT1), then cross the basolateral side of the colonocytes through SLC4A1/2 and MCT4 transporters, and are finally released into the bloodstream [58]. SCFAs interact with both intestinal and extraintestinal host cells through G protein-coupled receptors (GPCRs). Key receptors include free fatty acid receptors (FFARs)—specifically FFAR2 (also known as GPR43) and FFAR3 (GPR41), the butyrate-specific receptor GPR109A, and the acetate- and propionate-specific receptor OLFR78 [57]. SCFAs are metabolized into important intermediates such as acetyl-CoA and succinyl-CoA, which enter the tricarboxylic acid cycle and contribute to cellular energy production, lipogenesis, and gluconeogenesis [59]. In addition, SCFAs regulate gene expression by inhibiting histone deacetylases (HDACs) and activating G protein-coupled receptors, thereby modulating metabolic, inflammatory, and immune pathways [59]. Furthermore, SCFAs contribute to host metabolic homeostasis by enhancing intestinal barrier integrity, stimulating mucin production, regulating gut motility via enteroendocrine signaling, and reducing intestinal inflammation by suppressing pro-inflammatory cytokine production and promoting regulatory immune responses [57]. SCFAs impact immune system balance. They decrease pro-inflammatory signaling by inhibiting NF-κB activation and reducing the production of cytokines (e.g., IL-1β, IL-6, TNF-α) and molecules involved in immune response activation, such as MCP1 and VCAM1. At the same time, SCFAs increase anti-inflammatory responses by promoting macrophage polarization from M1 to M2 and increasing IL-10, immunoglobulin A, and regulatory T and B lymphocytes [57]. A study on atherosclerosis observed that levels of SCFA-producing bacteria, such as *R. intestinalis* and *F. prausnitzii*, were lower in patients than in healthy controls [60]. The protective effects of SCFAs on plaque stability are well-documented [48,54]. Specifically, SCFAs improve vascular health by inducing nitric oxide synthesis in endothelial cells, promoting vasodilation while also reducing inflammatory responses. Further, SCFAs can inhibit TMAO formation and diminish the translocation of deleterious microbial constituents, such as LPS, into the systemic circulation by improving intestinal barrier integrity. Collectively, all these mechanisms may contribute to the indirect stabilization of atherosclerotic plaques [61]. Administration of butyrate has been associated with stabilization of atherosclerotic plaques, characterized by increased fibrous cap thickness and decreased MMP-2 activity, the latter of which is implicated in the degradation of extracellular matrix components within plaques of ApoE^−/−^ mice. These effects were mediated by reduced oxidative stress in situ, which contributed to the anti-inflammatory and plaque-stabilizing effects of butyrate treatment [62,63].

While the beneficial effects of butyrate on the stability of atherosclerotic plaques are well documented in pre-clinical studies, translating these findings into clinical practice remains a formidable challenge, primarily due to its limited bioavailability and rapid metabolic degradation. The development of a pro-drug, SerBut, has successfully overcome these problems by enhancing bioavailability and preserving the anti-inflammatory properties of butyrate. In particular, in ApoE^−/−^ mice, this pro-drug significantly inhibited plaque progression, reduced monocyte content in the aorta, and reduced circulating levels of inflammatory cytokines, thereby improving plaque stability compared to sodium butyrate [64]. Propionate has been shown to reduce aortic atherosclerotic lesion area in ApoE^−/−^ mice fed a high-fat diet by increasing regulatory Treg cell numbers and local anti-inflammatory IL-10 levels, which suppress the expression of Niemann-Pick C1-like 1 (Npc1l1), a major intestinal cholesterol transporter. Propionate’s ability to increase IL-10 levels suggests that it may help stabilize atherosclerotic plaques by modulating inflammatory responses [65]. Oral acetate administration inhibited arteriosclerotic plaque progression and reduced necrotic core formation in the aortic sinus of ApoE^−/−^ mice compared with vehicle-treated mice by reducing macrophage proliferation, inflammatory cytokine release, and ROS production in macrophages through GPR43-mediated AMPK activation, thereby contributing to plaque stabilization [66].

#### 3.2.3. Phenylacetylglutamine (PAGln)

Phenylacetylglutamine (PAGln) is a newly identified gut-microbiome-related metabolite reported for the first time in a study by Nemet and collaborators [67]. PAGln is generated through microbial conversion of dietary phenylalanine into phenylacetic acid, which is subsequently conjugated with glutamine in the host liver. Using untargeted metabolomics in over 1000 subjects, the authors identified PAGln (derived from dietary phenylalanine) as a plasma microbially generated metabolite strongly associated with major adverse cardiovascular events over a 3-year period. Studies using microbial colonization in both healthy subjects and germ-free mice have revealed that PAGln may enhance platelet activation and contribute to thrombosis. Gain-of-function and loss-of-function analyses established that PAGln acted through adrenergic receptors. Later, other studies demonstrated that PAGln is not only a prothrombotic metabolite but also a potential contributor to atherosclerosis progression and even plaque vulnerability through activation of inflammatory signaling, oxidative stress, and endothelial dysfunction [68,69]. Very recently, a clinical study showed that patients with CAD had significantly higher serum PAGln concentrations, particularly among those with vulnerable plaques, compared with those with stable plaques. The authors speculated that PAGln, derived from gut microbiota dysbiosis, might destabilize plaques by interfering with lipid metabolism, promoting foam cell formation, and activating systemic inflammation [70].

### 3.3. Bile Acid (BA) Regulation

Bile acids (BAs) are amphipathic metabolites produced primarily by hepatocytes from cholesterol via the enzyme cholesterol 7α-hydroxylase (CYP7A1). In smaller amounts, BAs are also synthesized in extrahepatic tissues through sterol 27-hydroxylase (CYP27A1). The products of these enzymes are known as primary BAs, specifically cholic acid (CA) and chenodeoxycholic acid (CDCA) in humans. These primary BAs are then conjugated by bile acid-CoA synthetase and bile acid-CoA:amino acid N-acyltransferase, with taurine or glycine to form bile salts that are stored in the gall bladder and released into the duodenum following food intake [71,72]. Nearly all BAs are reabsorbed in the terminal portion of the small intestine and recycled via enterohepatic circulation. However, the remaining BAs, approximately 5%, enter the colon, where they are metabolized by the gut microbiota into secondary BAs—deoxycholic acid and lithocholic acid—via dehydroxylation, and are then passively reabsorbed [73]. Once reabsorbed, BAs bind specific receptors such as farnesoid × receptor (FXR), vitamin D receptor (VDR), constitutive androstane receptor (CAR), pregnane × receptor (PXR), and certain G-protein-coupled receptors (GPCRs) such as TGR5, sphingosine-1-phosphate receptor 2 (S1PR2), muscarinic acetylcholine receptor M3 (M3) and Mas-related G protein-coupled receptor member X4 (MRGPRX4). These receptors modulate lipid, glucose, and BA metabolism and influence the immune system [73]. BAs impact the abundance, diversity, and metabolic activity of the gut microbiota [74]. In particular, they play a significant role in the formation of vulnerable atherosclerotic plaques through several mechanisms [54]. BAs function as signaling molecules by activating receptors such as FXR and GPBAR1/TGR5, which regulate downstream pathways including cAMP, PPAR, and PI3K-AKT. Through these signaling cascades, BAs modulate cholesterol metabolism, inflammatory responses, macrophage polarization, and endothelial function, thereby influencing plaque composition and stability [75]. Alterations in bile acid composition may promote vascular inflammation, oxidative stress, and lipid accumulation, thereby contributing to plaque progression and destabilization. Moreover, the interaction between BAs and the gut microbiota diversifies the bile acid pool, thereby affecting atherosclerosis development and promoting plaque instability [76]. Probiotics such as *Bifidobacterium animalis* subsp. *lactis F1–7* have been shown to modulate bile acid metabolism, reducing aortic plaque accumulation by downregulating FXR and altering the gut microbiota composition. Specifically, this probiotic reduced lithocholic acid levels and regulated the FXR/FGF15/CYP7A1 pathway, thereby promoting bile acid homeostasis and improving lipid metabolism [77].

### 3.4. Lipopolysaccharide (LPS) Release

LPS is a component of the outer membrane of Gram-negative bacteria and plays an important role in the development and progression of atherosclerosis [76]. The immune system recognizes LPS as a pathogen-associated molecular pattern (PAMP), initiating inflammatory responses integral to atherogenesis. The interaction of LPS with TLR4 on various cell types, including those in the vascular system, activates NF-κB and other transcription factors, initiating a cascade of inflammatory processes that contribute to the development of atherosclerotic plaques [78,79,80,81]. This pathway showcases an essential molecular relationship linking gut-derived signals to vascular inflammation, which drives plaque destabilization. Interestingly, several studies have demonstrated that LPS can also promote the formation of vulnerable plaques [82,83]. Specifically, in an animal model with a vulnerable plaque phenotype, LPS and physical stress (electric foot shock and noise stimulation) were used to trigger plaque disruption. Both psychological stress and LPS stimulation markedly diminished the thickness of the fibrous cap, reduced collagen deposition, and increased lipid and macrophage accumulation within atherosclerotic plaques. Furthermore, the synergistic effect of stress and LPS stimulation induced the highest plaque disruption rate, most likely because of enhanced expression of pro-inflammatory cytokines and MMPs [82].

A recent study showed that LPS induced the formation of atherosclerotic plaques and increased plaque vulnerability in the carotid arteries of hypercholesterolemic Yucatan microswine. Findings from ultrasound, angiography, and optical coherence tomography revealed that localized LPS administration following intimal injury reduced fibrous cap thickness. Furthermore, these data were corroborated by in vitro studies showing increased expression of inflammatory mediators and plaque-vulnerability markers in vascular smooth muscle and endothelial cells treated with LPS, compared with untreated cells [83].

### 3.5. Immune Modulation

The gut microbiota regulates both innate and adaptive immune responses [84,85], and this modulation promotes foam cell formation, increases lipid accumulation, and supports necrotic core growth, which helps the formation and progression of atherosclerotic plaques. In fact, the gut microbiota triggers the production of cytokines and chemokines, which boost the differentiation and activation of certain immune cells, and affect atherosclerotic plaque formation and stability. As a result, the immune system indirectly controls plaque stability by altering plaque composition and initiating inflammatory responses [85,86]. Certain immune cell groups influenced by the gut microbiota, both nearby and throughout the body, have pro-atherogenic effects. These include Th1 cells and some B cell types, such as B2-Follicular B (FOB) cells [87,88]. These cells exacerbate inflammation, which induces plaque growth. Additionally, immune system imbalances can alter the microbiota, thereby increasing inflammation and disrupting plaque balance.

### 3.6. Inflammatory Pathways

Dysbiosis, defined as an imbalance within the gut microbiota, results in the depletion of beneficial bacterial populations, including SCFA-producing species such as *F. prausnitzii* and *Roseburia* spp., which normally preserve epithelial barrier integrity, concomitantly allowing the proliferation of potentially pro-inflammatory microorganisms, including members of the Enterobacteriaceae family and other Gram-negative bacteria. Dietary habits, antibiotic exposure, lifestyle factors, aging, and host genetics are among the major determinants influencing the balance between beneficial and potentially harmful microbial communities. These events result in a leaky gut and, as a consequence, bacterial components such as LPS can translocate into the bloodstream, triggering inflammatory pathways that worsen plaque instability [54,89]. This systemic inflammation activates vascular endothelium, promotes monocyte recruitment, and fuels macrophage polarization toward a pro-inflammatory M1 phenotype inside plaques [90].

In particular, the circulating inflammatory mediators and microbial metabolites act on plaques to increase endothelial permeability, therefore supporting further monocyte entry. Furthermore, these factors induce apoptosis in VSMCs, reducing fibrous cap thickness, whereas they facilitate expansion of the necrotic core via lipid accumulation and impaired efferocytosis. Importantly, these inflammatory species activate macrophages and smooth muscle cells in the vasculature, which subsequently release MMP-2, MMP-9, and cathepsins, thereby promoting extracellular matrix breakdown. These changes shift a stable, fibrotic plaque into a vulnerable plaque, consequently heightening its risk of rupture.

## 4. Mechanisms by Which Gut-Microbiota-Derived Metabolites Influence Plaque Stability

### 4.1. Modulation of Macrophage Polarization

Macrophages are central players in inflammation and remodeling of atherosclerotic plaques, as well as in inducing an inflammatory response that can differentially affect plaque stability [91]. Originally, macrophages were divided into two subclasses (M1, promoting inflammation, and M2, associated with anti-inflammatory and reparative functions) [92]. Nevertheless, single-cell and spatial transcriptomic technologies have identified several different subsets of macrophages in atherosclerotic plaques. Recent spatial and single-cell analyses indicate that distinct macrophage subsets accumulate in distinct regions of the atherosclerotic plaque, where metabolite availability can induce phenotypic switches in macrophages [93]. For instance, an enrichment in 5-hydroxyindoleacetic acid in the fibrous cap, revealed by a study evaluating the spatial distribution of metabolites in human atherosclerotic plaque, might explain the presence of pro-inflammatory macrophages in this part of unstable plaques [93]. Among these metabolic compounds, the gut metabolite indole-3-acetic acid (IAA), a tryptophan-derived molecule, is particularly interesting. The gastrointestinal tract is the main site of IAA production via tryptophan-dependent metabolic pathways [94]. Several commensal bacterial genera contribute to IAA synthesis, including *Clostridium*, *Roseburia*, *Faecalibacterium*, *Lactobacillus reuteri*, *Bifidobacterium*, *Bacteroides fragilis*, and *Bacteroides thetaiotaomicron* [95]. In a recent study, it was demonstrated that IAA inhibited activation of the TLR4/MyD88/NF-κB signaling cascade in M1 macrophages, thereby reducing the secretion of pro-inflammatory cytokines and promoting a transition towards the M2 phenotype [96]. This shift in macrophage polarization resulted in decreased vascular inflammation, enhanced collagen deposition, and improved stability of atherosclerotic plaques. In addition to from tryptophan metabolites, butyrate and other SCFAs function as significant regulators of macrophage polarization. In this regard, butyrate promotes M2 macrophage polarization in both in vitro and in vivo experimental settings, partly by enhancing H3K9 acetylation and subsequent STAT6-mediated transcriptional activity [97]. In atherosclerotic ApoE^−/−^ mice, dietary butyrate supplementation was associated with reduced macrophage adhesion and migration and increased collagen deposition in the lesion, resulting in increased plaque stability [62].

### 4.2. Inhibition of Macrophage Efferocytosis

Cells that undergo programmed death, such as apoptosis, pyroptosis, or ferroptosis, can be engulfed by specialized phagocytes; in atherosclerotic plaques, these are primarily macrophages. This process, known as efferocytosis, is crucial for slowing lesion progression and contributing to plaque stability. In advanced plaques, characterized by persistent inflammation, phagocytes may become less effective, leading to impaired efferocytosis and, therefore, to the advancement of atherosclerotic lesions and the formation of unstable plaques [98,99]. Several mechanisms underlie defective efferocytosis in atherosclerotic lesions. Reduced activity of the TAM family receptor MerTK, a key efferocytic receptor, decreases apoptotic cell uptake and promotes necrotic core expansion [100].

Additionally, apoptotic cells in advanced lesions frequently express CD47, a “do not eat me” signal that inhibits their recognition and clearance by macrophages [101]. Interestingly, efferotabolism represents a central metabolic reprogramming in macrophages that enables the resolution of inflammation and enhances clearance activity. Failure of this axis perpetuates unresolved inflammation and represents a feature of advanced atherosclerotic plaques [102,103]. Recent evidence indicates that metabolites produced by the gut microbiota can also influence efferocytosis. Notably, the branched-chain amino acid leucine has been identified as a negative regulator [104]. The addition of leucine to an atherogenic diet in atherosclerosis-prone ApoE^−/−^ mice impaired apoptotic cell clearance within the atherosclerotic plaque, leading to plaque progression. These effects were mediated above a threshold by leucine-induced MTORC1 signaling, which impaired autophagy/mitophagy, led to the accumulation of dysfunctional mitochondria, increased ROS levels, and triggered apoptosis. Therefore, leucine, by sustaining inflammation, contributes to the expansion of the necrotic core and to plaque destabilization [104].

### 4.3. Regulation of Intraplaque Metalloprotease Activity

MMPs are a family of zinc-dependent endoproteases responsible for tissue remodeling and the degradation of extracellular matrix components. MMPs play a key role in the progression of atherosclerotic lesions and plaque destabilization, as their activity can lead to the formation of a thin fibrous cap and a large necrotic core [105].

Among them, MMP-2 and MMP-9 are the most extensively studied, and their elevated expression has been consistently associated with vulnerable plaques and adverse cardiovascular outcomes [106,107]. Experimental evidence indicates that gut microbiota may influence intraplaque MMP activity. In a recent study, administration of *Dendrobium huoshanense* (DHP), a traditional medicinal herb, significantly reduced the upregulation of MMP-2 and MMP-9 by promoting gut microbiota balance, thereby inhibiting the progression of arteriosclerotic plaques [108].

In a fecal microbiota transplantation model, ApoE^−/−^ mice colonized with microbiota from patients with acute coronary syndrome developed plaques with larger necrotic cores, thinner fibrous caps, reduced α-SMA^+^ smooth muscle cells, and increased MMP2 activity. In contrast, microbiota from patients with chronic coronary syndrome were associated with more stable plaques. These effects were linked to an unfavorable microbial metabolic profile [109].

## 5. Experimental Models and Clinical Evidence

### 5.1. Animal Models of Atherosclerosis and Microbiota

Accumulating pre-clinical evidence indicates that the gut microbiota plays a role in modulating atherosclerosis development and therapeutic responses. The sodium–glucose cotransporter 2 inhibitor empagliflozin is known for its anti-atherosclerotic effects in different experimental models [110,111,112]. A recent study showed that these effects could be transmitted through the gut microbiota [113]. Specifically, fecal microbiota transplantation (FMT) from ApoE^−/−^ mice treated with the sodium–glucose cotransporter 2 inhibitor (SGLT2i) empagliflozin significantly attenuated atherosclerosis progression in ApoE^−/−^ recipient mice fed a high-fat diet. According to the authors, these effects were mediated by alterations in microbiota composition. These alterations were associated with reduced fecal BA levels and modulation of intestinal FXR signaling, which is involved in cholesterol metabolism and reverse cholesterol transport, thereby contributing to anti-atherosclerotic effects. Further, FMT–empagliflozin recipient mice showed lower levels of valeric acid, which has been shown to promote intestinal inflammation. Accordingly, the authors suggested that empagliflozin might attenuate systemic inflammation and thereby markedly reduce atherosclerotic lesions by lowering levels of bacteria associated with valeric acid production. In another atherosclerosis-prone model, the C1q/TNF-related protein 9 knockout (CTRP9-KO) mouse, gut dysbiosis (characterized by increased *Bacteroidetes* and reduced *Firmicutes* abundance) promoted plaque formation, whereas FMT from wild-type donors markedly attenuated lesion progression [114]. Similarly, a pro-inflammatory microbiota derived from Casp1^−/−^ donors, characterized by reduced abundance of SCFA-producing taxa such as *Akkermansia*, *Christensenellaceae*, *Clostridium*, and *Odoribacter* and lower intestinal SCFA levels, increased plaque burden by nearly 30% in Ldlr^−/−^ recipients and reduced circulating SCFAs [115]. Beyond microbial transfer, new approaches such as cyclic peptide treatment have been used to remodel the microbiota in Ldlr^−/−^ mice, resulting in lower plasma cholesterol and reduced atherosclerosis; these benefits were abolished after antibiotic administration, confirming a microbiota-dependent mechanism [116]. Inulin orally administered in ApoE^−/−^ mice fed a high-fat diet for 12 weeks reduced plaque formation in these mice. These effects were associated with increased gut microbial richness and diversity. Specifically, this improvement correlated with the maintenance of lipid metabolic homeostasis, a reduction in localized and systemic inflammation, and the protection of intestinal barrier integrity, all hallmarks of stable plaques [117]. Together, these findings underscore the importance of the gut–vascular axis as a central mediator of atherosclerosis and highlight the potential of microbiota-targeted strategies, including dietary supplementation, as adjunctive therapies for cardiovascular disease.

### 5.2. In Vivo Studies on Microbiota-Mediated Plaque Stability/Vulnerability

**Figure 3:**
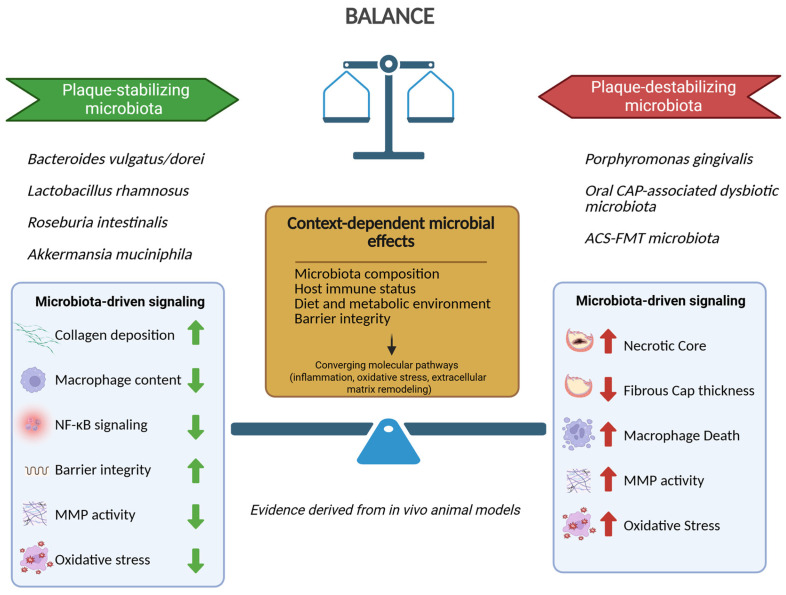
In vivo microbiota effects on atherosclerotic plaque stability versus instability.

Emerging in vivo studies highlight the influence of gut microbiota on atherosclerotic plaque vulnerability, not only on plaque burden [48] (Table 1 and Figure 3).

In vivo studies indicate that distinct gut microbial populations differentially modulate atherosclerotic plaque phenotypes through context-dependent influences on shared molecular pathways. Microbiota that stabilize plaques, such as *Bacteroides vulgatus/dorei*, *Lactobacillus rhamnosus*, *Roseburia intestinalis* and *Akkermansia muciniphila*, are associated with reduced inflammation, diminished NF-κB signaling, attenuated matrix metalloproteinase (MMP) activity, augmented collagen deposition, and enhanced integrity of the intestinal barrier. Conversely, microbiota that destabilize plaques, including *Porphyromonas gingivalis*, chronic apical periodontitis (CAP)-associated dysbiotic microbiota characterized by alterations in taxa such as *Akkermansia*, *Allobaculum*, *Sutterella*, and reduced *Lactobacillus*, as well as microbiota from acute coronary syndrome (ACS) patients in fecal microbiota transplantation (FMT) studies, enhance inflammation, increase oxidative stress, macrophage death, MMP activation, necrotic core enlargement and thinning of the fibrous cap. Critically, these results depend on context and are influenced by the microbiota’s composition, the host’s immune status, dietary factors, and the metabolic milieu. Although there are taxonomic differences, both stabilizing and destabilizing microbial communities converge on a limited set of molecular pathways, including inflammation, oxidative stress, and extracellular matrix remodeling, which ultimately govern the stability or vulnerability of plaques. This evidence is substantiated by findings from in vivo animal models.

Yoshida et al. found that oral administration of *Bacteroides vulgatus* and *Bacteroides dorei* significantly reduced plaque inflammation in mice prone to atherosclerosis through microbiota-dependent pathways. Supplementation with these bacteria reduced gut microbial LPS production, thereby lowering systemic endotoxemia and attenuating TLR4-driven immune responses. These effects were linked to fewer macrophages and CD4^+^ T cells within atherosclerotic lesions, along with a shift toward a more immunoregulatory profile, as evidenced by lower levels of pro-inflammatory cytokines, including IL-2, IL-4, IL-6, IL-17A, interferon-γ, and TNF-α. Collectively, these findings point to processes that promote plaque stabilization, such as reduced inflammatory signaling and immune cell infiltration, which are factors that determine plaque vulnerability. Thus, the data offer in vivo evidence that specific gut microbial taxa can impact features of atherosclerotic plaques that differentiate stable from rupture-prone forms [80].

Similar anti-atherogenic effects have been reported for other intestinal bacteria, including *Lactobacillus rhamnosus* (*L. rhamnosus*), a Gram-positive, facultative anaerobic probiotic that has been extensively studied for its immunomodulatory and barrier-protective properties [121,124]. Early evidence showed its indirect anti-atherogenic effects: supplementation with *L. rhamnosus* significantly reduced high-fat diet-induced atherosclerotic plaque size in ApoE^−/−^ mice, exerted by reducing cholesterol crystals in the plaques and circulating E-selectin, ICAM-1, and VCAM1 [124]. However, more recent in vivo studies demonstrated that treatment with different *L. rhamnosus* strains decreased atherosclerotic lesion size in ApoE^−/−^ mice by restraining oxidative stress, inflammation, and metalloprotease activity within the plaque [121,122]. Part of these effects were probably mediated by activation of the Nrf2/HO-1 pathway, leading to reduced oxidative stress and downstream inflammatory signaling in the vascular wall [122]. Additionally, analysis of atherosclerotic plaques from patients with symptomatic atherosclerotic disease and asymptomatic atherosclerotic plaques from a control group showed enrichment of *L. rhamnosus* DNA in stable, compared with unstable, carotid plaques [10], suggesting that administration of *L. rhamnosus* may increase atherosclerotic plaque stability.

Importantly, recent studies suggest that bacteria may also influence plaque vulnerability through shared metabolic and inflammatory mechanisms rather than by directly altering plaque structure. In this context, *Eggerthella*, and specifically *E. lenta*, has emerged as a taxon inversely associated with inflammatory markers. In a very recent study, blocking the EphA2 receptor with ALW-II-41-27 reduced atherosclerotic plaques and improved lipid profiles by remodeling the gut microbiota in ApoE^-/-^ mice fed a high-fat diet. The gut microbiota analysis showed enrichment of beneficial bacteria, such as *Eggerthella* and *Lactobacillus*, accompanied by enhanced secondary bile acid (SBA) production [118]. It is noteworthy that SBAs exhibited statistically significant negative correlations with aortic plaque areas, confirming that the treatment efficacy of ALW-II-41-27 against atherosclerosis was, at least in part, associated with its manipulation of the gut microbiome and bile acid metabolism. Further, SBAs are ligands of the G protein-coupled receptor TGR5 whose activation attenuated atherosclerosis in Ldlr^−/−^Tgr5^+/+^ mice, whereas these protective effects were absent in Ldlr^−/−^Tgr5^−/−^ mice, supporting a direct role of TGR5 signaling in reducing intraplaque inflammation and plaque macrophage content [125].

Beyond specific probiotic species, increasing attention has been paid to gut bacteria that produce SCFAs, particularly butyrate, due to their significant effects on host metabolism, immune regulation, and intestinal barrier function. In a study by Kasahara and colleagues, it was demonstrated that *Roseburia intestinalis* might alter the susceptibility and progression of atherosclerosis by increasing cecal butyrate concentration in germ-free ApoE^−/−^ mice fed a diet high in plant polysaccharides. To confirm the role of butyrate, direct intestinal administration of butyrate reduced endotoxemia and hindered the development of atherosclerosis. Interestingly, in both cases, the authors showed that mice developed atherosclerotic lesions characterized by reduced macrophage content and increased collagen content, suggesting that colonization with butyrate-producing bacteria promoted the stability of atherosclerotic plaques [120].

Different studies have demonstrated that increases in *A. muciniphila* abundance correlate with reduced atherosclerosis and more stable plaques (as evidenced by higher collagen content and reduced inflammation within the plaque) in multiple mouse models and following probiotic interventions [119,126,127,128]. Nevertheless, it should be noted that different studies have reported context-dependent pro-inflammatory roles of this bacterium in conditions other than CAD, including Salmonella typhimurium infection, inflammatory bowel disease, and multiple sclerosis [129,130,131,132]. Specifically, in multiple sclerosis, elevated levels have been linked to pro-inflammatory immune signatures, with a lower *Bifidobacterium adolescentis*-to-*Akkermansia* ratio emerging as a disease hallmark [131,132]. These findings show that while *A. Muciniphila* usually supports mucosal health, it may likewise contribute to immune activation or epithelial erosion in inflamed or immunocompromised hosts [133].

In contrast to beneficial bacteria such as *A. muciniphila*, *Roseburia intestinalis*, *L. rhamnosus*, and other SCFA-producing taxa, which are associated with reduced inflammation and more stable plaque phenotypes, compelling in vivo evidence shows that certain oral pathobionts substantially contribute to atherosclerotic plaque vulnerability. Among these, *Porphyromonas gingivalis*, a Gram-negative periodontal pathogen, is one of the most important examples of a bacterium associated with plaque destabilization. A very recent investigation involving rabbits and ApoE^−/−^ mice has shown that recurrent exposure to *P. gingivalis* led to the formation of plaques characterized by large necrotic cores, increased macrophage necroptosis, elevated oxidative stress, and other indicators of plaque instability [123]. The results expand on previous research showing that chronic oral infection with *P. gingivalis* accelerated inflammatory atherosclerosis in the innominate artery of ApoE^−/−^ mice, a vascular site predisposed to the development of human-like advanced plaques that exhibit rupture and intraplaque hemorrhage [134]. Collectively, these results provide direct evidence that *P. gingivalis* not only accelerates atherogenesis but also actively promotes the transition toward a more rupture-prone plaque phenotype.

Additionally, studies focused on infections by individual species are integrated by models of chronic apical periodontitis (CAP), which strengthen the theory of a direct association between oral microbial ecosystems and plaque instability. In ApoE^−/−^ mice, the induction of CAP using pulp tissue infected with *P. gingivalis* resulted in an exacerbation of atherosclerotic burden, systemic and vascular inflammation, gut dysbiosis and compromised intestinal barrier integrity [135]. *P. gingivalis*, a well-recognized periodontal pathogen, has been shown to promote chronic inflammation, endothelial dysfunction, and microbial dysbiosis, thereby contributing to atherogenesis. Recent investigations have further elucidated these findings, indicating that CAP triggered by *P. gingivalis* correlates not only with increased lesion size but also with plaque-vulnerability features, including larger necrotic cores and enhanced inflammation [136]. Collectively, these findings suggest that chronic oral infection driven by *P. gingivalis* and the associated microbial dysbiosis may exacerbate vascular inflammation and promote the development of unstable plaque phenotypes.

### 5.3. Clinical Trials Investigating Microbiota and Atherosclerosis

In an early study, Mitra and colleagues found significant differences in gut microbiota composition between patients with stable versus unstable plaques [10] (Table 2). Specifically, they compared carotid plaques obtained from patients with repeated transient ischemic attacks or minor stroke (considered symptomatic/unstable plaques) with asymptomatic plaques collected at autopsy from individuals who died from causes not related to atherosclerotic disease.

In contrast, Lindskog Jonsson and colleagues showed the presence of bacterial DNA in carotid atherosclerotic plaques from patients undergoing endarterectomy. However, they did not find differences in microbial abundance and composition between asymptomatic and symptomatic plaques, suggesting that microbial DNA profiles might not explain differences in plaque vulnerability [137]. Nevertheless, some researchers propose that bacterial DNA may activate macrophages and trigger the innate immune system via TLR2 and TLR4, which in turn may influence plaque stability [139].

Two other clinical studies highlighted the contribution of the microbiota of patients with chronic and acute coronary syndromes to plaque instability [11,12]. Both studies identified specific bacteria associated with both favorable and unfavorable coronary plaque characteristics. Nakajima and colleagues demonstrated that some bacteria associated with vulnerable plaque features were also significantly correlated with elevated inflammatory or prothrombotic biomarkers, suggesting a potential role for the gut microbiota in the development of vulnerable plaques. Interestingly, Pisano and colleagues compared the gut microbiota with the coronary plaque microbiota and found that, while the former exhibited a highly heterogeneous composition, with significant levels of *Bacteroidetes* and *Firmicutes*, the latter was enriched in microbes with pro-inflammatory phenotypes, primarily *Proteobacteria*. Therefore, although the gut hosted a diverse community, the plaque environment selected for specific, potentially harmful bacteria. Nevertheless, neither study established a definitive pathogenic role of the microbiota in the context of coronary instability.

More recently, additional clinical evidence has further linked gut microbiota dysbiosis to acute coronary events. In particular, a recent investigation elucidated that patients with acute coronary syndrome display unique signatures of gut microbiota that correlate with specific dietary patterns and systemic inflammatory biomarkers, thereby corroborating the hypothesis that dysbiosis could play a role in enhancing plaque susceptibility [140]. Consistent with these observations, Ahmad and colleagues reported differential abundances in specific gut bacterial groups and related metabolites between patients with chronic stable angina and those with acute coronary syndrome, which may be linked to the inflammatory, metabolomic, and lipidomic profiles of these patients. Accordingly, they detected reduced levels of propionate and butyrate, as well as elevated serum levels of the inflammatory markers sCD14 and sCD163, in patients with acute coronary syndrome compared to patients with chronic stable angina and healthy controls, suggesting that gut microbial dysbiosis may contribute to inflammatory processes associated with plaque destabilization [138].

Interestingly, clinical studies are beginning to test whether modulation of the gut microbiota can translate into vascular benefits in humans. In a randomized, double-blind, placebo-controlled trial, supplementation with *Lactobacillus plantarum 299v* in patients with stable CAD improved endothelial function and reduced systemic inflammatory markers, including IL-8, IL-12 and leptin, while leaving traditional cardiometabolic risk factors (cholesterol, fasting glucose, BMI) unchanged. Interestingly, plasma collected after supplementation enhanced endothelium-dependent vasodilation in resistance arteries, pointing to a role for gut-derived metabolites in mediating these vascular effects [141]. A synbiotic trial in overweight patients with type 2 diabetes and CAD showed significant reductions in high-sensitivity C-reactive protein (hs-CRP) and oxidative stress markers, and improvements in nitric oxide bioavailability. However, carotid intima–media thickness (CIMT) did not change during the study period [142]. Apart from interventional trials, observational data also support a correlation between microbiota composition and vascular health. In people with HIV, specific microbial species were associated with subclinical atherosclerosis progression measured by carotid intima–media thickness measurements. *Agathobacter* and *Ruminococcus 2* were associated with disease progression, while non-progression was consistently associated with *Prevotella 7* [143]. Collectively, these results indicate that targeted modulation of the gut microbiome can improve systemic inflammation and vascular function, strengthening the concept of a microbiota–vascular axis in human atherosclerosis.

### 5.4. Challenges and Limitations in Translating Microbiota Research to Clinical Practice

Despite rapid advances, translating microbiome research findings linking the gut microbiota to atherosclerosis and plaque instability into effective clinical treatments remains challenging. Firstly, gut microbial composition is highly individualized because it depends on diet, medication use, habits, and host genetics, all of which make it difficult to identify a universal microbial signature associated with plaque vulnerability [144]. Causality is also difficult to establish: many human studies remain observational, and therefore specific microbial taxa or metabolites may be consequences rather than causes of disease. Another limitation is methodological heterogeneity (differences in sampling techniques, sequencing technologies, and bioinformatics methodologies), which reduces the possibility of comparing different studies [145]. Importantly, much of the existing data stems from animal models, which restrict the capability to generalize findings to human populations. Finally, the complex network of interactions between microbial metabolites and host signaling pathways is only partially understood and, for this reason, it remains very difficult to design precise microbiota-targeted therapies [146]. Nevertheless, growing evidence suggests that microbiota-modulating interventions, including natural compounds such as berberine, which have exhibited antiatherogenic effects in clinical and pre-clinical studies [147], may represent promising approaches for reducing cardiovascular risk by modulating the gut–heart axis. However, further mechanistic and clinical studies are required.

## 6. Conclusions

The gut microbiota represents an important regulator of atherosclerosis, affecting not only plaque extension but also its phenotype and stability. Experimental studies have shown that metabolites derived from the gut microbiota can influence processes underlying plaque vulnerability. These include inflammation, immune cell recruitment, extracellular matrix remodeling, endothelial dysfunction and plaque cellular composition. For example, SCFAs are associated with protective effects. In contrast, metabolites such as TMAO, PAGln, BAs and LPS have been shown to induce plaque vulnerability. Importantly, several in vivo studies have shown that certain microbial species, including Bacteroidetes, Lactobacillus, and butyrate-producing bacteria, stabilize plaques, whereas bacteria associated with dysbiosis promote plaque instability.

Nevertheless, these effects are not universally applicable and may exhibit context-dependent variability, as demonstrated by Akkermansia muciniphila, whose influence is contingent on the host’s metabolic and immunological status. Despite these improvements in the field, translating microbiota research into clinical practice remains very challenging.

Major limitations include the high interindividual variability of the gut microbiota, methodological heterogeneity among studies, the predominance of observational clinical evidence, and the limited translatability of animal models to human disease. Furthermore, causal relationships between specific microbial signatures, microbial metabolites, and plaque vulnerability remain incompletely understood.

Future studies should also move beyond descriptive microbiota profiling based on 16S rRNA sequencing and increasingly integrate whole-genome metagenomics and metabolomics approaches. These technologies provide species-level resolution and functional insights into microbiota-derived metabolites and host–microbiota interactions, thereby improving the identification of causal mechanisms and potential therapeutic targets. However, their widespread clinical application is still limited by cost and technical complexity. Further studies are needed to deepen our understanding of host–microbiota interactions and context-dependent effects. Further research is also necessary to clarify the microbiota’s causal role in plaque instability. This information is needed to develop personalized therapeutic strategies useful for both the prevention and treatment of CAD, as well as for the treatment of atherosclerotic plaques.

## Figures and Tables

**Table 1:** Experimental evidence linking microbiota to plaque stability.

Bacterium/Microbial Taxa	Model	Plaque-Related Endpoints	Effect on Plaque Phenotype	Ref.
*Eggerthella lenta*	ApoE^−/−^ mice	Reduced inflammatory markers and improved lipid profiles	Reduces atherosclerotic plaque size	[118]
*Akkermansia muciniphila*	ApoE^−/−^ mice	Improved gut barrier integrity and reduced systemic inflammation and macrophage infiltration	Associated with reduced atherosclerotic lesion formation and improved plaque stability	[119]
*Roseburia intestinalis*	Germ-free ApoE^−/−^ mice	Reduced macrophage infiltration and increased collagen deposition	Promotes plaque stabilization	[120]
*Bacteroides vulgatus*/*B. dorei*	ApoE^−/−^ mice	Reduced vascular inflammation and lesion development through reduced LPS production and lower levels of pro-inflammatory cytokines	Protective effect against atherosclerosis	[80]
*Lactobacillus rhamnosus*	ApoE^−/−^ mice	Reduced inflammatory cytokine levels, oxidative stress and metalloprotease activity within the plaque	Associated with decreased atherosclerotic lesion size and increased atherosclerotic plaque stability	[121,122]
*Porphyromonas gingivalis*	ApoE^−/−^ mice/rabbit models	Increased macrophage infiltration and necrotic core formation	Promotes plaque destabilization	[123]

**Table 2:** Clinical evidence linking microbiota and plaque vulnerability.

Population	Sample Analyzed	Plaque/Clinical Classification	Main Findings	Clinical Implication	Ref.
Patients undergoing carotid endarterectomy	Carotid plaque microbiome	Stable vs. unstable plaques	Distinct bacterial taxa were detected in unstable plaques	Plaque-associated microbiota may contribute to plaque instability	[10]
Patients undergoing carotid endarterectomy	Carotid plaque tissue (bacterial DNA)	Symptomatic vs. asymptomatic plaques	Bacterial DNA was detected in plaques, but no significant differences in microbial load or composition were observed	Presence of plaque bacteria does not necessarily correlate with plaque instability	[137]
Patients with carotid atherosclerosis	Coronary plaque bacterial DNA	Stable vs. unstable plaques	Bacterial DNA was more frequently detected in unstable plaques	Microbial components may contribute to plaque destabilization	[12]
Patients with coronary artery disease	Gut microbiota sequencing	Stable CAD vs. ACS	Specific gut microbial signatures were associated with ACS and vulnerable plaque features	Gut microbiota composition may reflect plaque vulnerability	[11]
Patients with stable angina, ACS, and controls	Gut microbiota sequencing and serum metabolomics	Stable CAD vs. ACS	ACS patients showed microbiota alterations, reduced SCFAs, and increased inflammatory markers	Microbiota–metabolite alterations may contribute to plaque destabilization	[138]
Patients with carotid atherosclerosis undergoing endarterectomy	Gut microbiota sequencing and carotid plaque analysis	Stable vs. unstable plaques	Distinct gut microbial profiles were associated with unstable plaques and inflammatory taxa enrichment	Gut microbial dysbiosis may represent a biomarker of plaque vulnerability	[70]

## Data Availability

No new data were created or analyzed in this study. Data sharing is not applicable to this article.

## References

[1] Libby P. (2021). The changing Nature of atherosclerosis: What we thought we knew, what we think we know, and what we have to learn. Eur. Heart J..

[2] Jebari-Benslaiman S., Galicia-García U., Larrea-Sebal A., Olaetxea J.R., Alloza I., Vandenbroeck K., Benito-Vicente A., Martín C. (2022). Pathophysiology of Atherosclerosis. Int. J. Mol. Sci..

[3] Ruigrok R.A.A.A., Weersma R.K., Vich Vila A. (2023). The emerging role of the small intestinal microbiota in human health and disease. Gut Microbes.

[4] Thursby E., Juge N. (2017). Introduction to the human gut microbiota. Biochem. J..

[5] Govender P., Ghai M. (2025). Population-specific differences in the human microbiome: Factors defining the diversity. Gene.

[6] Gupta V.K., Paul S., Dutta C. (2017). Geography, Ethnicity or Subsistence-Specific Variations in Human Microbiome Composition and Diversity. Front. Microbiol..

[7] Escobar C., Aldeguer X., Vivas D., Manzano Fernández S., Gonzalez Caballero E., Garcia Martín A., Barrios V., Freixa-Pamias R. (2025). The gut microbiota and its role in the development of cardiovascular disease. Expert Rev. Cardiovasc. Ther..

[8] Laryushina Y., Samoilova-Bedych N., Turgunova L., Kozhakhmetov S., Alina A., Suieubayev M., Mukhanbetzhanov N. (2024). Alterations of the Gut Microbiome and TMAO Levels in Patients with Ulcerative Colitis. J. Clin. Med..

[9] Shin Y., Han S., Kwon J., Ju S., Choi T.G., Kang I., Kim S.S. (2023). Roles of Short-Chain Fatty Acids in Inflammatory Bowel Disease. Nutrients.

[10] Mitra S., Drautz-Moses D.I., Alhede M., Maw M.T., Liu Y., Purbojati R.W., Yap Z.H., Kushwaha K.K., Gheorghe A.G., Bjarnsholt T. (2015). In silico analyses of metagenomes from human atherosclerotic plaque samples. Microbiome.

[11] Nakajima A., Mitomo S., Yuki H., Araki M., Seegers L.M., McNulty I., Lee H., Kuter D., Ishibashi M., Kobayashi K. (2022). Gut Microbiota and Coronary Plaque Characteristics. J. Am. Heart Assoc..

[12] Pisano E., Bugli F., Severino A., Pedicino D., Paroni Sterbini F., Martini C., De Maio F., Vinci R., Sacconi A., Canonico F. (2023). Microbial signature of plaque and gut in acute coronary syndrome. Sci. Rep..

[13] Task A., Members F., Byrne R.A., Ireland C., Rossello X., Coughlan J.J., Ireland T.F.C., Barbato E., Berry C., Kingdom U. (2023). 2023 ESC Guidelines for the management of acute coronary syndromes: Developed by the task force on the management of acute coronary syndromes of the European Society of Cardiology (ESC). Eur. Heart J..

[14] Liu W., Wang L., Ou J., Peng D., Zhang Y., Chen W., Wang Y. (2025). Gut Microbiota Metabolites and Chronic Diseases: Interactions, Mechanisms, and Therapeutic Strategies. Int. J. Mol. Sci..

[15] Murray C.J.L. (2022). The Global Burden of Disease Study at 30 years. Nat. Med..

[16] Björkegren J.L.M., Lusis A.J. (2022). Atherosclerosis: Recent developments. Cell.

[17] Vourakis M., Mayer G., Rousseau G. (2021). The Role of Gut Microbiota on Cholesterol Metabolism in Atherosclerosis. Int. J. Mol. Sci..

[18] Mosalmanzadeh N., Pence B.D. (2024). Oxidized Low-Density Lipoprotein and Its Role in Immunometabolism. Int. J. Mol. Sci..

[19] He Y., Liu T. (2023). Oxidized low-density lipoprotein regulates macrophage polarization in atherosclerosis. Int. Immunopharmacol..

[20] Barreto J., Karathanasis S.K., Remaley A., Sposito A.C. (2021). Role of LOX-1 (Lectin-Like Oxidized Low-Density Lipoprotein Receptor 1) as a Cardiovascular Risk Predictor: Mechanistic Insight and Potential Clinical Use. Arter. Thromb. Vasc. Biol..

[21] Gimbrone M.A., García-Cardeña G. (2016). Endothelial Cell Dysfunction and the Pathobiology of Atherosclerosis. Circ. Res..

[22] Vergallo R., Crea F. (2020). Atherosclerotic Plaque Healing. N. Engl. J. Med..

[23] Puylaert P., Zurek M., Rayner K.J., De Meyer G.R.Y., Martinet W. (2022). Regulated Necrosis in Atherosclerosis. Arter. Thromb. Vasc. Biol..

[24] Kong P., Cui Z.Y., Huang X.F., Zhang D.D., Guo R.J., Han M. (2022). Inflammation and atherosclerosis: Signaling pathways and therapeutic intervention. Signal Transduct. Target. Ther..

[25] Doran A.C., Ozcan L., Cai B., Zheng Z., Fredman G., Rymond C.C., Dorweiler B., Sluimer J.C., Hsieh J., Kuriakose G. (2017). CAMKIIγ suppresses an efferocytosis pathway in macrophages and promotes atherosclerotic plaque necrosis. J. Clin. Investig..

[26] Bentzon J.F., Otsuka F., Virmani R., Falk E. (2014). Mechanisms of plaque formation and rupture. Circ. Res..

[27] Hu J., Van den Steen P.E., Sang Q.X.A., Opdenakker G. (2007). Matrix metalloproteinase inhibitors as therapy for inflammatory and vascular diseases. Nat. Rev. Drug Discov..

[28] Lina D., Paoli G., Menozzi A., Ardissino D. (2006). Instabilità di placca ed eventi aterotrombotici: Parte 2. Opportunità terapeutiche. Trends Med..

[29] Kasikara C., Doran A.C., Cai B., Tabas I. (2018). The role of non-resolving inflammation in atherosclerosis. J. Clin. Investig..

[30] Hafiane A. (2019). Vulnerable Plaque, Characteristics, Detection, and Potential Therapies. J. Cardiovasc. Dev. Dis..

[31] Basatemur G.L., Jørgensen H.F., Clarke M.C.H., Bennett M.R., Mallat Z. (2019). Vascular smooth muscle cells in atherosclerosis. Nat. Rev. Cardiol..

[32] Leung S.W.S., Shi Y. (2022). The glycolytic process in endothelial cells and its implications. Acta Pharmacol. Sin..

[33] Chen P.-Y., Qin L., Li G., Wang Z., Dahlman J.E., Malagon-Lopez J., Gujja S., Cilfone N.A., Kauffman K.J., Sun L. (2019). Endothelial TGF-β signalling drives vascular inflammation and atherosclerosis. Nat. Metab..

[34] Souilhol C., Serbanovic-Canic J., Fragiadaki M., Chico T.J., Ridger V., Roddie H., Evans P.C. (2020). Endothelial responses to shear stress in atherosclerosis: A novel role for developmental genes. Nat. Rev. Cardiol..

[35] Wang J., Wu R., Hua Y., Ling S., Xu X. (2023). Naringenin ameliorates vascular senescence and atherosclerosis involving SIRT1 activation. J. Pharm. Pharmacol..

[36] Fahed A.C., Jang I.K. (2021). Plaque erosion and acute coronary syndromes: Phenotype, molecular characteristics and future directions. Nat. Rev. Cardiol..

[37] Eshghjoo S., Kim D.M., Jayaraman A., Sun Y., Alaniz R.C. (2022). Macrophage Polarization in Atherosclerosis. Genes.

[38] Grootaert M.O.J., Bennett M.R. (2021). Vascular smooth muscle cells in atherosclerosis: Time for a re-assessment. Cardiovasc. Res..

[39] Newman A.A.C., Serbulea V., Baylis R.A., Shankman L.S., Bradley X., Alencar G.F., Owsiany K., Deaton R.A., Karnewar S., Shamsuzzaman S. (2021). Multiple cell types contribute to the atherosclerotic lesion fibrous cap by PDGFRβ and bioenergetic mechanisms. Nat. Metab..

[40] Zhai M., Gong S., Luan P., Shi Y., Kou W., Zeng Y., Shi J., Yu G., Hou J., Yu Q. (2022). Extracellular traps from activated vascular smooth muscle cells drive the progression of atherosclerosis. Nat. Commun..

[41] Kolaczkowska E., Kubes P. (2013). Neutrophil recruitment and function in health and inflammation. Nat. Rev. Immunol..

[42] Silvestre-Roig C., Braster Q., Wichapong K., Lee E.Y., Teulon J.M., Berrebeh N., Winter J., Adrover J.M., Santos G.S., Froese A. (2019). Externalized histone H4 orchestrates chronic inflammation by inducing lytic cell death. Nature.

[43] Ionita M.G., van den Borne P., Catanzariti L.M., Moll F.L., de Vries J.-P.P.M., Pasterkamp G., Vink A., de Kleijn D.P.V. (2010). High neutrophil numbers in human carotid atherosclerotic plaques are associated with characteristics of rupture-prone lesions. Arter. Thromb. Vasc. Biol..

[44] Drechsler M., Megens R.T.A., van Zandvoort M., Weber C., Soehnlein O. (2010). Hyperlipidemia-triggered neutrophilia promotes early atherosclerosis. Circulation.

[45] Zhang X., Kang Z., Yin D., Gao J. (2023). Role of neutrophils in different stages of atherosclerosis. Innate Immun..

[46] Silvestre-Roig C., Braster Q., Ortega-Gomez A., Soehnlein O. (2020). Neutrophils as regulators of cardiovascular inflammation. Nat. Rev. Cardiol..

[47] Bakogiannis C., Sachse M., Stamatelopoulos K., Stellos K. (2019). Platelet-derived chemokines in inflammation and atherosclerosis. Cytokine.

[48] Zheng S., Liu Z., Liu H., Lim J.Y., Li D.W.H., Zhang S., Luo F., Wang X., Sun C., Tang R. (2024). Research development on gut microbiota and vulnerable atherosclerotic plaque. Heliyon.

[49] Shen X., Li L., Sun Z., Zang G., Zhang L., Shao C., Wang Z. (2021). Gut Microbiota and Atherosclerosis-Focusing on the Plaque Stability. Front. Cardiovasc. Med..

[50] Li X.S., Obeid S., Klingenberg R., Gencer B., Mach F., Räber L., Windecker S., Rodondi N., Nanchen D., Muller O. (2017). Gut microbiota-dependent trimethylamine N-oxide in acute coronary syndromes: A prognostic marker for incident cardiovascular events beyond traditional risk factors. Eur. Heart J..

[51] Schiattarella G.G., Sannino A., Toscano E., Giugliano G., Gargiulo G., Franzone A., Trimarco B., Esposito G., Perrino C. (2017). Gut microbe-generated metabolite trimethylamine-N-oxide as cardiovascular risk biomarker: A systematic review and dose-response meta-analysis. Eur. Heart J..

[52] Ding L., Chang M., Guo Y., Zhang L., Xue C., Yanagita T., Zhang T., Wang Y. (2018). Trimethylamine-N-oxide (TMAO)-induced atherosclerosis is associated with bile acid metabolism. Lipids Health Dis..

[53] Liu C., Li Z., Song Z., Fan X., Shao H., Schönke M., Boon M.R., Rensen P.C.N., Wang Y. (2022). Choline and butyrate beneficially modulate the gut microbiome without affecting atherosclerosis in APOE*3-Leiden.CETP mice. Atherosclerosis.

[54] Alexandrescu L., Suceveanu A.P., Stanigut A.M., Tofolean D.E., Axelerad A.D., Iordache I.E., Herlo A., Nelson Twakor A., Nicoara A.D., Tocia C. (2024). Intestinal Insights: The Gut Microbiome’s Role in Atherosclerotic Disease: A Narrative Review. Microorganisms.

[55] Koay Y.C., Chen Y.-C., Wali J.A., Luk A.W.S., Li M., Doma H., Reimark R., Zaldivia M.T.K., Habtom H.T., Franks A.E. (2021). Plasma levels of trimethylamine-N-oxide can be increased with “healthy” and “unhealthy” diets and do not correlate with the extent of atherosclerosis but with plaque instability. Cardiovasc. Res..

[56] Cheng J., Cheng M., Sinha S., Cely I., Gladkikh S., Han M.T., Zhang G., Zhou Z., Chugh R., Ahn I.S. (2025). Trimethylamine-N-Oxide Affects Cell Type-Specific Pathways and Networks in Mouse Aorta to Promote Atherosclerotic Plaque Vulnerability. Arter. Thromb. Vasc. Biol..

[57] Mukhopadhya I., Louis P. (2025). Gut microbiota-derived short-chain fatty acids and their role in human health and disease. Nat. Rev. Microbiol..

[58] Fusco W., Lorenzo M.B., Cintoni M., Porcari S., Rinninella E., Kaitsas F., Lener E., Mele M.C., Gasbarrini A., Collado M.C. (2023). Short-Chain Fatty-Acid-Producing Bacteria: Key Components of the Human Gut Microbiota. Nutrients.

[59] Sukkar A.H., Lett A.M., Frost G., Chambers E.S. (2019). Regulation of energy expenditure and substrate oxidation by short-chain fatty acids. J. Endocrinol..

[60] Jie Z., Xia H., Zhong S.-L., Feng Q., Li S., Liang S., Zhong H., Liu Z., Gao Y., Zhao H. (2017). The gut microbiome in atherosclerotic cardiovascular disease. Nat. Commun..

[61] Wen X., Zhong R., Dang G., Xia B., Wu W., Tang S., Tang L., Liu L., Liu Z., Chen L. (2022). Pectin supplementation ameliorates intestinal epithelial barrier function damage by modulating intestinal microbiota in lipopolysaccharide-challenged piglets. J. Nutr. Biochem..

[62] Aguilar E.C., Leonel A.J., Teixeira L.G., Silva A.R., Silva J.F., Pelaez J.M.N., Capettini L.S.A., Lemos V.S., Santos R.A.S., Alvarez-Leite J.I. (2014). Butyrate impairs atherogenesis by reducing plaque inflammation and vulnerability and decreasing NFκB activation. Nutr. Metab. Cardiovasc. Dis..

[63] Aguilar E.C., Santos L.C.D., Leonel A.J., de Oliveira J.S., Santos E.A., Navia-Pelaez J.M., da Silva J.F., Mendes B.P., Capettini L.S.A., Teixeira L.G. (2016). Oral butyrate reduces oxidative stress in atherosclerotic lesion sites by a mechanism involving NADPH oxidase down-regulation in endothelial cells. J. Nutr. Biochem..

[64] Beckman T.N., Volpatti L.R., Norton de Matos S., Slezak A.J., Reda J.W., Weinstock A., Ziolkowski L., Turk A., Budina E., Cao S. (2025). A prometabolite strategy inhibits cardiometabolic disease in an ApoE^−/−^ murine model of atherosclerosis. JCI Insight.

[65] Haghikia A., Zimmermann F., Schumann P., Jasina A., Roessler J., Schmidt D., Heinze P., Kaisler J., Nageswaran V., Aigner A. (2022). Propionate attenuates atherosclerosis by immune-dependent regulation of intestinal cholesterol metabolism. Eur. Heart J..

[66] Wada T., Senokuchi T., Shi Y., Furusho T., Morita Y., Sarie M., Hanatani S., Fukuda K., Ishii N., Matsumura T. (2025). Orally administrated acetate inhibits atherosclerosis progression through AMPK activation via GPR43 in plaque macrophages. Atherosclerosis.

[67] Nemet I., Saha P.P., Gupta N., Zhu W., Romano K.A., Skye S.M., Cajka T., Mohan M.L., Li L., Wu Y. (2020). A Cardiovascular Disease-Linked Gut Microbial Metabolite Acts via Adrenergic Receptors. Cell.

[68] Fu H., Kong B., Zhu J., Huang H., Shuai W. (2023). Phenylacetylglutamine increases the susceptibility of ventricular arrhythmias in heart failure mice by exacerbated activation of the TLR4/AKT/mTOR signaling pathway. Int. Immunopharmacol..

[69] Fang C., Zuo K., Jiao K., Zhu X., Fu Y., Zhong J., Xu L., Yang X. (2022). PAGln, an Atrial Fibrillation-Linked Gut Microbial Metabolite, Acts as a Promoter of Atrial Myocyte Injury. Biomolecules.

[70] You X., Gao B. (2025). Association between Intestinal Flora Metabolites and Coronary Artery Vulnerable Plaque Characteristics in Coronary Heart Disease. Br. J. Hosp. Med..

[71] Chiang J.Y.L. (2013). Bile acid metabolism and signaling. Compr. Physiol..

[72] Fleishman J.S., Kumar S. (2024). Bile acid metabolism and signaling in health and disease: Molecular mechanisms and therapeutic targets. Signal Transduct. Target. Ther..

[73] Collins S.L., Stine J.G., Bisanz J.E., Okafor C.D., Patterson A.D. (2023). Bile acids and the gut microbiota: Metabolic interactions and impacts on disease. Nat. Rev. Microbiol..

[74] Li Y., Tang R., Leung P.S.C., Gershwin M.E., Ma X. (2017). Bile acids and intestinal microbiota in autoimmune cholestatic liver diseases. Autoimmun. Rev..

[75] Cheng X., Zhang R., Qi X., Wang H., Gao T., Zheng L., Qiao M., Li Y., Gao S., Chen J. (2024). Metabolomics and network pharmacology exploration of the effects of bile acids on carotid atherosclerosis and potential underlying mechanisms. Front. Endocrinol..

[76] Tonch-Cerbu A.K., Boicean A.G., Stoia O.M., Teodoru M. (2025). Gut Microbiota-Derived Metabolites in Atherosclerosis: Pathways, Biomarkers, and Targets. Int. J. Mol. Sci..

[77] Liang X., Zheng X., Wang P., Zhang H., Ma Y., Liang H., Zhang Z. (2024). Bifidobacterium animalis subsp. lactis F1-7 Alleviates Lipid Accumulation in Atherosclerotic Mice via Modulating Bile Acid Metabolites to Downregulate Intestinal FXR. J. Agric. Food Chem..

[78] Bowman J.D., Surani S., Horseman M.A. (2017). Endotoxin, Toll-like Receptor-4, and Atherosclerotic Heart Disease. Curr. Cardiol. Rev..

[79] Sieve I., Ricke-Hoch M., Kasten M., Battmer K., Stapel B., Falk C.S., Leisegang M.S., Haverich A., Scherr M., Hilfiker-Kleiner D. (2018). A positive feedback loop between IL-1β, LPS and NEU1 may promote atherosclerosis by enhancing a pro-inflammatory state in monocytes and macrophages. Vasc. Pharmacol..

[80] Yoshida N., Emoto T., Yamashita T., Watanabe H., Hayashi T., Tabata T., Hoshi N., Hatano N., Ozawa G., Sasaki N. (2018). Bacteroides vulgatus and Bacteroides dorei Reduce Gut Microbial Lipopolysaccharide Production and Inhibit Atherosclerosis. Circulation.

[81] Carnevale R., Nocella C., Petrozza V., Cammisotto V., Pacini L., Sorrentino V., Martinelli O., Irace L., Sciarretta S., Frati G. (2018). Localization of lipopolysaccharide from Escherichia Coli into human atherosclerotic plaque. Sci. Rep..

[82] Ni M., Wang Y., Zhang M., Zhang P.F., Ding S.F., Liu C.X., Liu X.L., Zhao Y.X., Zhang Y. (2009). Atherosclerotic plaque disruption induced by stress and lipopolysaccharide in apolipoprotein E knockout mice. Am. J. Physiol. Heart Circ. Physiol..

[83] Nooti S., Rai V., Radwan M.M., Thankam F.G., Singh H., Chatzizisis Y.S., Agrawal D.K. (2023). Oxidized Low-density Lipoproteins and Lipopolysaccharides Augment Carotid Artery Plaque Vulnerability in Hypercholesterolemic Microswine. Cardiol. Cardiovasc. Med..

[84] Chistiakov D.A., Bobryshev Y.V., Kozarov E., Sobenin I.A., Orekhov A.N. (2015). Role of gut microbiota in the modulation of atherosclerosis-associated immune response. Front. Microbiol..

[85] Wang C., Deng H., Liu F., Yin Q., Xia L. (2022). Role of gut microbiota in the immunopathology of atherosclerosis: Focus on immune cells. Scand. J. Immunol..

[86] Giakomidi D., Ishola A., Nus M. (2025). Targeting gut microbiota to regulate the adaptive immune response in atherosclerosis. Front. Cardiovasc. Med..

[87] Liang W.L., Liao H.L., Liang B. (2023). Immune landscape and regulatory mechanisms in human atherosclerotic coronary plaques: Evidence from single-cell and bulk transcriptomics. Heliyon.

[88] Centa M., Jin H., Hofste L., Hellberg S., Busch A., Baumgartner R., Verzaal N.J., Lind Enoksson S., Perisic Matic L., Boddul S.V. (2019). Germinal Center-Derived Antibodies Promote Atherosclerosis Plaque Size and Stability. Circulation.

[89] Chakaroun R.M., Massier L., Kovacs P. (2020). Gut Microbiome, Intestinal Permeability, and Tissue Bacteria in Metabolic Disease: Perpetrators or Bystanders?. Nutrients.

[90] Ajoolabady A., Pratico D., Lin L., Mantzoros C.S., Bahijri S., Tuomilehto J., Ren J. (2024). Inflammation in atherosclerosis: Pathophysiology and mechanisms. Cell Death Dis..

[91] Libby P., Geng Y.J., Aikawa M., Schoenbeck U., Mach F., Clinton S.K., Sukhova G.K., Lee R.T. (1996). Macrophages and atherosclerotic plaque stability. Curr. Opin. Lipidol..

[92] Moore K.J., Tabas I. (2011). Macrophages in the pathogenesis of atherosclerosis. Cell.

[93] Seeley E.H., Liu Z., Yuan S., Stroope C., Cockerham E., Rashdan N.A., Delgadillo L.F., Finney A.C., Kumar D., Das S. (2023). Spatially Resolved Metabolites in Stable and Unstable Human Atherosclerotic Plaques Identified by Mass Spectrometry Imaging. Arter. Thromb. Vasc. Biol..

[94] Duca D.R., Glick B.R. (2020). Indole-3-acetic acid biosynthesis and its regulation in plant-associated bacteria. Appl. Microbiol. Biotechnol..

[95] Shaheen N., Miao J., Xia B., Zhao Y., Zhao J. (2025). Multifaceted Role of Microbiota-Derived Indole-3-Acetic Acid in Human Diseases and Its Potential Clinical Application. FASEB J..

[96] Liu W., Wang J., Yang H., Li C., Lan W., Chen T., Tang Y. (2025). The Metabolite Indole-3-Acetic Acid of Bacteroides Ovatus Improves Atherosclerosis by Restoring the Polarisation Balance of M1/M2 Macrophages and Inhibiting Inflammation. Adv. Sci..

[97] Ji J., Shu D., Zheng M., Wang J., Luo C., Wang Y., Guo F., Zou X., Lv X., Li Y. (2016). Microbial metabolite butyrate facilitates M2 macrophage polarization and function. Sci. Rep..

[98] Kojima Y., Weissman I.L., Leeper N.J. (2017). The Role of Efferocytosis in Atherosclerosis. Circulation.

[99] Li W., Huang Y., Liu J., Zhou Y., Sun H., Fan Y., Liu F. (2024). Defective macrophage efferocytosis in advanced atherosclerotic plaque and mitochondrial therapy. Life Sci..

[100] Thorp E., Cui D., Schrijvers D.M., Kuriakose G., Tabas I. (2008). Mertk receptor mutation reduces efferocytosis efficiency and promotes apoptotic cell accumulation and plaque necrosis in atherosclerotic lesions of apoe^−/−^ mice. Arter. Thromb. Vasc. Biol..

[101] Shu L.X., Cao L.L., Guo X., Wang Z.B., Wang S.Z. (2024). Mechanism of efferocytosis in atherosclerosis. J. Mol. Med..

[102] Yurdagul A. (2021). Metabolic Consequences of Efferocytosis and its Impact on Atherosclerosis. Immunometabolism.

[103] Kumar D., Pandit R., Yurdagul A. (2023). Mechanisms of continual efferocytosis by macrophages and its role in mitigating atherosclerosis. Immunometabolism.

[104] Zhang X., Ajam A., Liu Z., Peroumal D., Khan S.R., Razani B. (2025). Leucine accelerates atherosclerosis through dose-dependent MTOR activation in macrophages. Autophagy.

[105] Olejarz W., Łacheta D., Kubiak-Tomaszewska G. (2020). Matrix Metalloproteinases as Biomarkers of Atherosclerotic Plaque Instability. Int. J. Mol. Sci..

[106] Samah N., Ugusman A., Hamid A.A., Sulaiman N., Aminuddin A. (2023). Role of Matrix Metalloproteinase-2 in the Development of Atherosclerosis among Patients with Coronary Artery Disease. Mediat. Inflamm..

[107] Li T., Li X., Feng Y., Dong G., Wang Y., Yang J. (2020). The Role of Matrix Metalloproteinase-9 in Atherosclerotic Plaque Instability. Mediat. Inflamm..

[108] Zheng S.J., Gao X.Y., Diao X.H., Chen N.D. (2025). Dendrobium huoshanense improves atherosclerosis in high-fat-induced ApoE mice by regulating gut microbiota and serum metabolite profiles. Phytomedicine.

[109] Nieri R., Foglio E., De Maio F., Severino A., Masucci L., Aiello A.D., Grimaldi M.C., Gervasoni J., Santoni D., Pedicino D. (2024). Abstract 4143104: Atherosclerotic features of plaque instability are transmitted via gut microbial transplantation. Circulation.

[110] Pennig J., Scherrer P., Gissler M.C., Anto-Michel N., Hoppe N., Füner L., Härdtner C., Stachon P., Wolf D., Hilgendorf I. (2019). Glucose lowering by SGLT2-inhibitor empagliflozin accelerates atherosclerosis regression in hyperglycemic STZ-diabetic mice. Sci. Rep..

[111] Han J.H., Oh T.J., Lee G., Maeng H.J., Lee D.H., Kim K.M., Choi S.H., Jang H.C., Lee H.S., Park K.S. (2017). The beneficial effects of empagliflozin, an SGLT2 inhibitor, on atherosclerosis in ApoE^−/−^ mice fed a western diet. Diabetologia.

[112] Liu Y., Xu J., Wu M., Xu B., Kang L. (2021). Empagliflozin protects against atherosclerosis progression by modulating lipid profiles and sympathetic activity. Lipids Health Dis..

[113] Hao H., Li Z., Qiao S.-Y., Qi Y., Xu X.-Y., Si J.-Y., Liu Y.-H., Chang L., Shi Y.-F., Xu B. (2023). Empagliflozin ameliorates atherosclerosis via regulating the intestinal flora. Atherosclerosis.

[114] Kim E.S., Yoon B.H., Lee S.M., Choi M., Kim E.H., Lee B.-W., Kim S.-Y., Pack C.-G., Sung Y.H., Baek I.-J. (2022). Fecal microbiota transplantation ameliorates atherosclerosis in mice with C1q/TNF-related protein 9 genetic deficiency. Exp. Mol. Med..

[115] Brandsma E., Kloosterhuis N.J., Koster M., Dekker D.C., Gijbels M.J.J., van der Velden S., Ríos-Morales M., van Faassen M.J.R., Loreti M.G., de Bruin A. (2019). A Proinflammatory Gut Microbiota Increases Systemic Inflammation and Accelerates Atherosclerosis. Circ. Res..

[116] Chen P.B., Black A.S., Sobel A.L., Zhao Y., Mukherjee P., Molparia B., Moore N.E., Aleman Muench G.R., Wu J., Chen W. (2020). Directed remodeling of the mouse gut microbiome inhibits the development of atherosclerosis. Nat. Biotechnol..

[117] Zhang K., Zeng Y., Li J., Huang Y., Zhang N., Gong Y., Xiao K., Chen J., Chen T., Qiu H. (2024). Inulin alleviates atherosclerosis through improving lipid metabolism, inflammation, and gut microbiota in ApoE-knockout mice: The short-chain is more efficacious. Front. Pharmacol..

[118] Lu C., Liu D., Wu Q., Zeng J., Xiong Y., Luo T. (2024). EphA2 blockage ALW-II-41-27 alleviates atherosclerosis by remodeling gut microbiota to regulate bile acid metabolism. npj Biofilms Microbiomes.

[119] Li J., Lin S., Vanhoutte P.M., Woo C.W., Xu A. (2016). Akkermansia Muciniphila Protects Against Atherosclerosis by Preventing Metabolic Endotoxemia-Induced Inflammation in Apoe^−/−^ Mice. Circulation.

[120] Kasahara K., Krautkramer K.A., Org E., Romano K.A., Kerby R.L., Vivas E.I., Mehrabian M., Denu J.M., Bäckhed F., Lusis A.J. (2018). Interactions between Roseburia intestinalis and diet modulate atherogenesis in a murine model. Nat. Microbiol..

[121] Fang Y., Chen H.-Q., Zhang X., Zhang H., Xia J., Ding K., Fang Z.-Y. (2019). Probiotic administration of lactobacillus rhamnosus GR-1 attenuates atherosclerotic plaque formation in ApoE^−/−^ mice fed with a high-fat diet. Eur. Rev. Med. Pharmacol. Sci..

[122] Liu Y., Bai Z., Yan R., Ma J., Wang L., Li Y., Liu Y., Ma H., Wang T., Yang L. (2025). Lactobacillus rhamnosus GG ameliorates atherosclerosis via suppression of oxidative stress and inflammation by reshaping the gut microbiota. Biochem. Biophys. Res. Commun..

[123] Huang X., Xie M., Wang Y., Lu X., Mei F., Zhang K., Yang X., Chen G., Yin Y., Feng G. (2025). Porphyromonas gingivalis aggravates atherosclerotic plaque instability by promoting lipid-laden macrophage necroptosis. Signal Transduct. Target. Ther..

[124] Chan Y.K., Brar M.S., Kirjavainen P.V., Chen Y., Peng J., Li D., Leung F.C.-C., El-Nezami H. (2016). High fat diet induced atherosclerosis is accompanied with low colonic bacterial diversity and altered abundances that correlates with plaque size, plasma A-FABP and cholesterol: A pilot study of high fat diet and its intervention with Lactobacillus rhamnosus GG (LGG) or telmisartan in ApoE^−/−^ mice. BMC Microbiol..

[125] Pols T.W.H., Nomura M., Harach T., Lo Sasso G., Oosterveer M.H., Thomas C., Rizzo G., Gioiello A., Adorini L., Pellicciari R. (2011). TGR5 activation inhibits atherosclerosis by reducing macrophage inflammation and lipid loading. Cell Metab..

[126] Xiao X., Wu Y., Jie Z., Lin L., Li Y., Hu W., Li Y., Zhong S. (2024). Akkermansia Muciniphila supplementation improves hyperlipidemia, cardiac function, and gut microbiota in high fat fed apolipoprotein E-deficient mice. Prostagland. Other Lipid Mediat..

[127] Gofron K., Berezowski A., Gofron M., Borówka M., Dziedzic M., Kazimierczak W., Kwiatkowski M., Gofron M., Nowaczyk Z., Małgorzewicz S. (2024). Akkermansia muciniphila—Impact on the cardiovascular risk, the intestine inflammation and obesity. Acta Biochim. Pol..

[128] Xu R., Zhang Y., Chen S., Zeng Y., Fu X., Chen T., Luo S., Zhang X. (2023). The role of the probiotic Akkermansia muciniphila in brain functions: Insights underpinning therapeutic potential. Crit. Rev. Microbiol..

[129] Seregin S.S., Golovchenko N., Schaf B., Chen J., Pudlo N.A., Mitchell J., Baxter N.T., Zhao L., Schloss P.D., Martens E.C. (2017). NLRP6 Protects Il10^−/−^ Mice from Colitis by Limiting Colonization of Akkermansia muciniphila. Cell Rep..

[130] Ganesh B.P., Klopfleisch R., Loh G., Blaut M. (2013). Commensal Akkermansia muciniphila exacerbates gut inflammation in Salmonella Typhimurium-infected gnotobiotic mice. PLoS ONE.

[131] Jangi S., Gandhi R., Cox L.M., Li N., von Glehn F., Yan R., Patel B., Mazzola M.A., Liu S., Glanz B.L. (2016). Alterations of the human gut microbiome in multiple sclerosis. Nat. Commun..

[132] Ghimire S., Lehman P.C., Aguilar Meza L.S., Shahi S.K., Hoang J., Olalde H., Paullus M., Cherwin C., Wang K., Gill C. (2025). Specific microbial ratio in the gut microbiome is associated with multiple sclerosis. Proc. Natl. Acad. Sci. USA.

[133] Tingler A.M., Engevik M.A. (2025). Breaking down barriers: Is intestinal mucus degradation by Akkermansia muciniphila beneficial or harmful?. Infect. Immun..

[134] Hayashi C., Viereck J., Hua N., Phinikaridou A., Madrigal A.G., Gibson F.C., Hamilton J.A., Genco C.A. (2011). Porphyromonas gingivalis accelerates inflammatory atherosclerosis in the innominate artery of ApoE deficient mice. Atherosclerosis.

[135] Gan G., Lu B., Zhang R., Luo Y., Chen S., Lei H., Li Y., Cai Z., Huang X. (2022). Chronic apical periodontitis exacerbates atherosclerosis in apolipoprotein E-deficient mice and leads to changes in the diversity of gut microbiota. Int. Endod. J..

[136] Gan G., Lin S., Luo Y., Zeng Y., Lu B., Zhang R., Chen S., Lei H., Cai Z., Huang X. (2024). Unveiling the oral-gut connection: Chronic apical periodontitis accelerates atherosclerosis via gut microbiota dysbiosis and altered metabolites in apoE^−/−^ Mice on a high-fat diet. Int. J. Oral Sci..

[137] Lindskog Jonsson A., Hållenius F.F., Akrami R., Johansson E., Wester P., Arnerlöv C., Bäckhed F., Bergström G. (2017). Bacterial profile in human atherosclerotic plaques. Atherosclerosis.

[138] Ahmad A.F., Caparrós-Martin J.A., Gray N., Lodge S., Wist J., Lee S., O’Gara F., Dwivedi G., Ward N.C. (2024). Gut microbiota and metabolomics profiles in patients with chronic stable angina and acute coronary syndrome. Physiol. Genom..

[139] Michelsen K.S., Arditi M. (2006). Toll-like receptor signaling and atherosclerosis. Curr. Opin. Hematol..

[140] López-Gálvez R., Orenes-Piñero E., Rivera-Caravaca J.M., Pérez-Sanz F., Ramos-Bratos M.P., Roca M.I., Mandaglio-Collados D., López-García C., Gil-Pérez P., Esteve-Pastor M.A. (2025). Microbial Insights: The Role of Diet in Modulating Gut Microbiota and Metabolites After Acute Coronary Syndrome. Mol. Nutr. Food Res..

[141] Malik M., Suboc T.M., Tyagi S., Salzman N., Wang J., Ying R., Tanner M.J., Kakarla M., Baker J.E., Widlansky M.E. (2018). Lactobacillus plantarum 299v Supplementation Improves Vascular Endothelial Function and Reduces Inflammatory Biomarkers in Men with Stable Coronary Artery Disease. Circ. Res..

[142] Farrokhian A., Raygan F., Soltani A., Tajabadi-Ebrahimi M., Sharifi Esfahani M., Karami A.A., Asemi Z. (2019). The Effects of Synbiotic Supplementation on Carotid Intima-Media Thickness, Biomarkers of Inflammation, and Oxidative Stress in People with Overweight, Diabetes, and Coronary Heart Disease: A Randomized, Double-Blind, Placebo-Controlled Trial. Probiotics Antimicrob. Proteins.

[143] Masiá M., García J.A., García-Abellán J., Padilla S., Fernández-González M., Agulló V., Gosalbes M.J., Ruíz-Pérez S., Mascarell P., Botella A. (2025). Distinct Gut Microbiota Signatures Associated with Progression of Atherosclerosis in People Living with Human Immunodeficiency Virus. J. Infect. Dis..

[144] Zhang Z.J., Lehmann C.J., Cole C.G., Pamer E.G. (2022). Translating Microbiome Research from and to the Clinic. Annu. Rev. Microbiol..

[145] Mirzayi C., Renson A., Zohra F., Elsafoury S., Geistlinger L., Kasselman L.J., Eckenrode K., van de Wijgert J., Genomic Standards Consortium, Massive Analysis and Quality Control Society (2021). Reporting guidelines for human microbiome research: The STORMS checklist. Nat. Med..

[146] Gilbert J.A., Azad M.B., Bäckhed F., Blaser M.J., Byndloss M., Chiu C.Y., Chu H., Dugas L.R., Elinav E., Gibbons S.M. (2025). Clinical translation of microbiome research. Nat. Med..

[147] Cao R.Y., Zhang Y., Feng Z., Liu S., Liu Y., Zheng H., Yang J. (2021). The Effective Role of Natural Product Berberine in Modulating Oxidative Stress and Inflammation Related Atherosclerosis: Novel Insights Into the Gut-Heart Axis Evidenced by Genetic Sequencing Analysis. Front. Pharmacol..

